# *In situ* forest with lycopsid trees bearing lobed rhizomorphs from the Upper Devonian of Lincheng, China

**DOI:** 10.1093/pnasnexus/pgae241

**Published:** 2024-06-15

**Authors:** Min Qin, Deming Wang, Le Liu, Lu Liu, Yi Zhou

**Affiliations:** College of Life Sciences, Linyi University, Linyi 276000, PR China; Key Laboratory of Stratigraphy and Palaeontology, Ministry of Natural Resources, Beijing 100037, PR China; Key Laboratory of Orogenic Belts and Crustal Evolution, Department of Geology, Peking University, Beijing 100871, PR China; School of Geoscience and surveying engineering, China University of Mining and Technology (Beijing), Beijing 100083, PR China; Department of Earth Sciences, National Natural History Museum of China, Beijing 100050, PR China; School of Life Sciences, Sun Yat-sen University, Guangzhou 510275, PR China

**Keywords:** fossil forest, tree lycopsid, rooting system, Upper Devonian

## Abstract

The earliest forests in the Devonian were reported from only four localities worldwide. The tree lycopsids, sometimes as the primary elements of Devonian forests, had evolved several types of rooting systems. In recent years, we found and excavated a Late Devonian (Famennian, 374–359 Ma) lycopsid forest from Zhejiang Province, China. The fossil forest occurs at seven locations of Lincheng Town of Changxing County and mainly consists of *in situ* small tree lycopsid (*Heliodendron longshanense* gen. et sp. nov.) stems usually connected to lobed cormose rhizomorphs. The four short lobes of each rhizomorph often branch once and bear roots arranged radially. Allometry is observed between the trunk diameter of *Heliodendron* and the length of its rhizomorphic lobes, indicating that the trunk develops later than the rhizomorph in tree lycopsid plants. The Devonian witnessed the transformation from clastic nonlycopsid dominated forests to Carboniferous swampy forests dominated by giant lycopsid trees. These trees form a multigenerational community, as shown by the *in situ* preserved stems at various levels within the same area due to frequent sedimentation events.

**Significance Statement**

Forests in their early evolutionary stages are rarely preserved as *in situ* fossils. With only four reported to date, any discovery of fossil forests is important for our understanding of the establishment of terrestrial ecosystems. We now describe a novel, well-preserved, and one of the earliest forests in Asia from the Upper Devonian of South China (374–359 Ma). The new forest consists of tree lycopsids with a special type of rooting system, providing new insights into the allometry, the sedimentary history of lycopsids, the structure of the early plant community, and the subsequent evolution of the giant trees in Carboniferous swamp forests.

## Introduction

Forests first appeared in the Devonian and had been reported from United States (i.e. Gilboa and Cairo forests near New York) (1, 2), Norway (Svalbard forest in Svalbard islands) (3), and China (Xinhang forest in Anhui Province) (4). Although these fossil forests had been studied in detail, it is still very difficult to find early forests on Earth. The lycopsids have the longest evolutionary history (Late Silurian to present) among vascular plant groups (5), and tree lycopsids represent the main components of Svalbard and Xinhang forests.

The rooting system plays a crucial role in facilitating the growth of individual tree lycopsid, providing essential mechanical support and efficient nutrient absorption. The narrow elongate and often dichotomous organs responsible for absorption and anchoring are referred to as roots, while the structure to which the roots are attached is known as the rhizomorph (6). The Devonian tree lycopsids may have presented all types of their belowground rooting systems, i.e. repeatedly dichotomized root, cormose rhizomorph (bulb-shaped and unbranched base), lobed cormose rhizomorph, and stigmarian rhizomorph (with four dichotomized axes) (3, 4, 7–10). Most of the giant tree lycopsids in Late Carboniferous (Pennsylvanian) coal swamps possessed the enormous stigmarian rhizomorph (11), while some smaller tree lycopsids displayed (lobed) cormose rhizomorph (12).

In recent years, a new fossil lycopsid forest was found and excavated from the Upper Devonian (Famennian) Wutong Formation in Lincheng Town, Changxing County, Zhejiang Province, China. Based on this discovery, we discuss the classification of rooting system and allometry in early tree lycopsids, the transition of early forests, and the preservation of Lincheng forest.

## Materials and methods

### Materials

Fossils described here include tree lycopsid *Heliodendron longshanense* gen. et sp. nov., possible Crinoidea fossils and some unidentifiable lycopsid trunks.

The fossils were preserved as impressions, compressions, and casts. All specimens are housed in the Department of Geology, Peking University, Beijing, China.

### Methods

The strata exposed in the field work area have been studied in previous work (13) and supported by geological maps (China National Digital Geological Map spatial database). Excavator was utilized initially to remove the overlying strata, and a big quadrat (5 m × 3 m) was subsequently established using nails, ropes, and marker pen. Manual excavation was then conducted in each small quadrat. Photos were taken to document newly excavated quadrats and fossils in them. Original data including tree position, diameter (the base of the trunk we can see, excluding the basal expanded area), length of rhizomorph lobes, and the depth of rooting systems (from the horizontal extension to the deepest portion of the root) were measured and recorded in the field book.

**Fig. 1.**
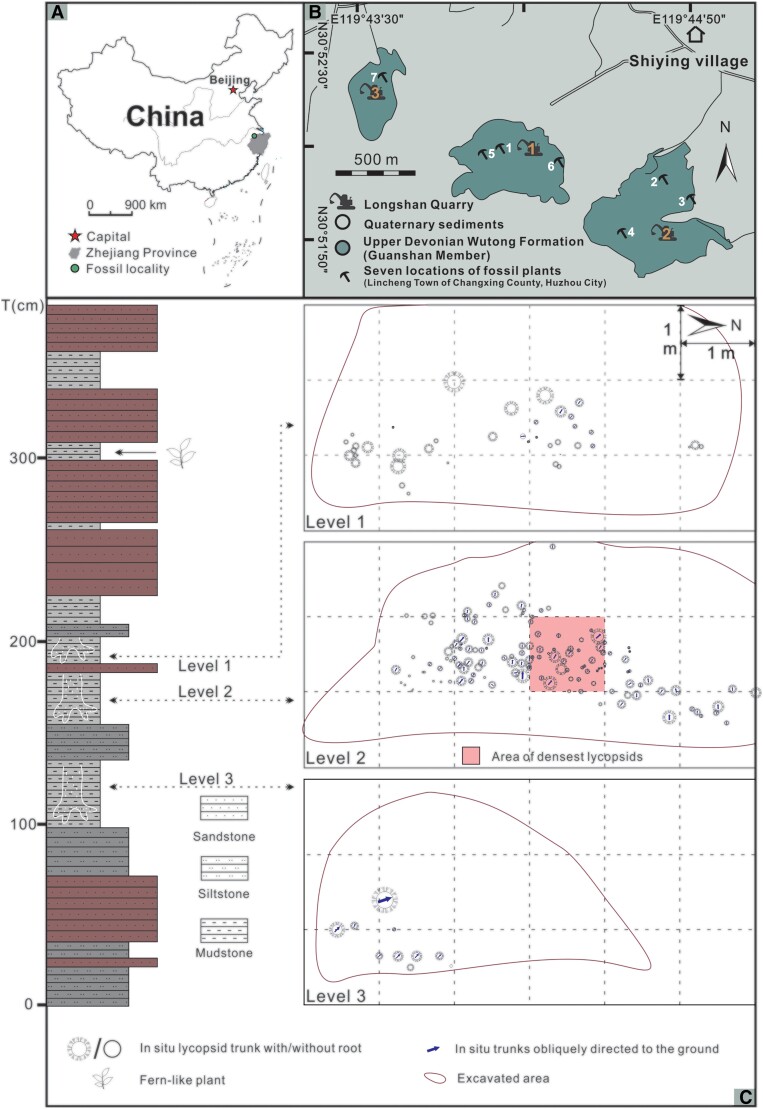
Location, stratigraphy and distribution of fossil plants. A) Map showing locality of Lincheng fossil lycopsid forest (in northwest part of Zhejiang Province). B) Seven locations (1–7) of *in situ* fossil plants in three Longshan quarries (1–3) near Shiying village, Lincheng Town, Changxing County of Zhejiang Province. C) Stratum of the Upper Devonian Wutong Formation (Guanshan Member) in location 1, and the distribution of fossil lycopsid (*Heliodendron*) in mudstone of three levels (levels 1–3, including quadrat); the diameters of *Heliodendron* are to the scale. The pink area has been expanded in SI-2 Fig. S4. The color of the left log corresponds to the hue of the rock. T (cm): thickness (centimeters).

**Fig. 2.**
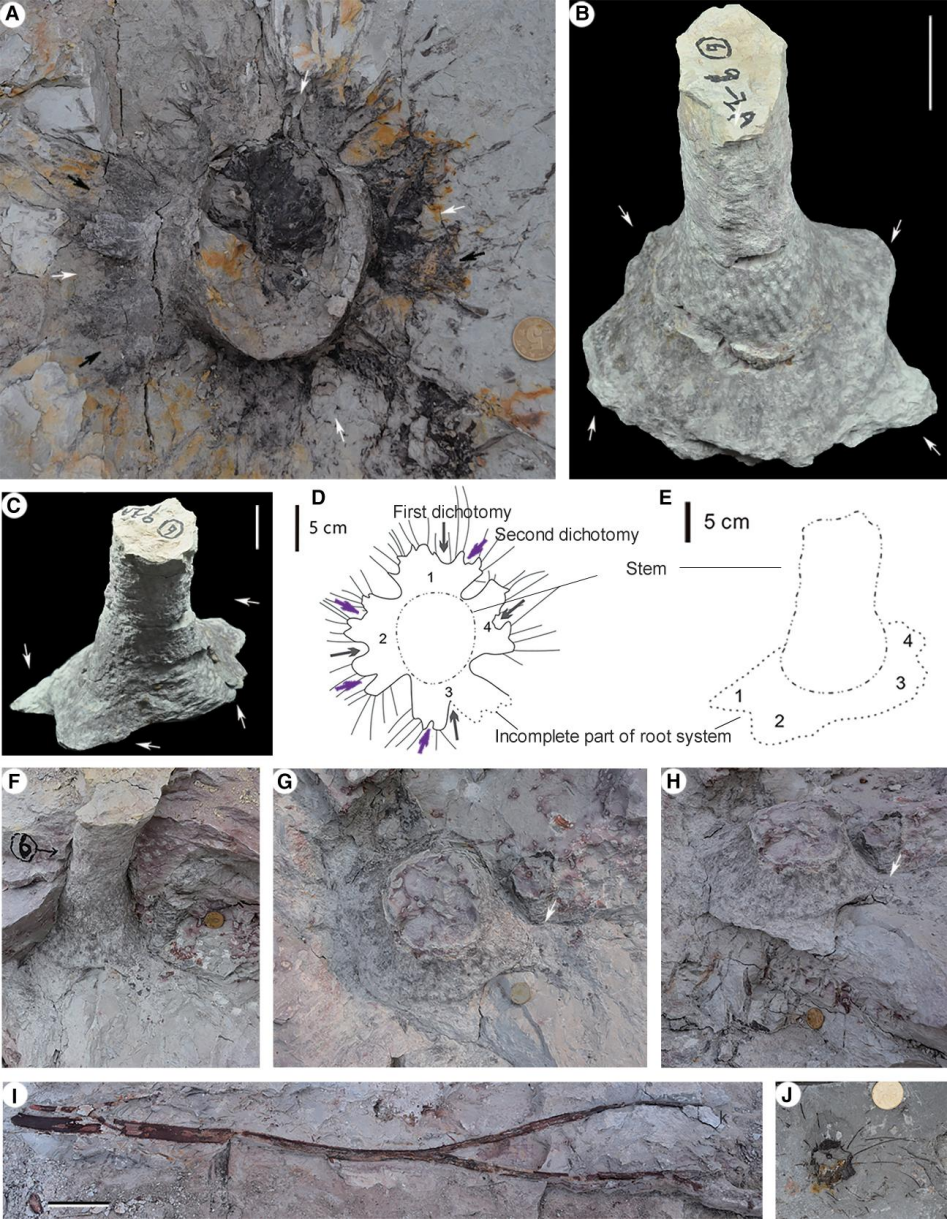
Individuals of lycopsid at location 1. A) Top view of a rooting system with short rhizomorph lobes (arrows) bearing roots (this axcavated root is originally connected with the stem in SI-1 Fig. S10A–F. White arrows indicating the first dichotomy and black arrows indicating the second dichotomy. Picture enlarged in SI-2 Fig. S1. B, C) Two opposite sides of the stump in Fig. 4A (arrow 6), same as in SI-1 Fig. S3C–G. Arrows indicating four lobes of the rhizomorph. PKUB17401. D) The line diagram of (C), showing four unequal and short rhizomorph lobes (numbers 1-4), with the first (gray arrows) and second dichotomy (purple arrows). E) The line diagram of (C), showing four unequal and short rhizomorph lobes (numbers 1-4). F–H) Serial excavation of stump in SI-1 Fig. S3B (arrow 1), the same stump as in (B, C). Arrows indicating the end of broken lobe. I) The longest trunk (lying on bedding plane) with dichotomy as in SI-1 Figs. S8A (black arrow) and S11A. J) A trunk across the bedding plane and with leaves, same as SI-1 Fig. S12C. PKUB17407. Scale bars = 2 cm (coin), 5 cm (B, C, D, E), 20 cm (I).

Steel needles and chisels were used to expose the morphology of plants, and digital cameras for photographs in the field and laboratory. All images were prepared and labeled in figures with Adobe Photoshop CC 2014. According to the data from Google map, Baidu map, geological map, and investigation at Longshan mine near Shiying village, Lincheng Town, Changxing County, Huzhou City, Zhejiang Province, China, the distributions and horizons of *in situ* fossil plants were provided in Fig. 1. Figure 1, the line diagram Figs. 2D, E and schematic diagram Fig. 7B were made by CorelDRAW X8, the 3-D reconstruction Fig. 7A was made by Plantfactory 2014. The diameters of stems of *in situ* lycopsids were analyzed through Microsoft Excel 2019.

## Results

The fossil forest occurs at seven locations in Longshan Mine (including three quarries) near Shiying village, Lincheng Town, Changxing County, Zhejiang Province, China (Figs. 1A, B). The *in situ* plants were buried at quarries 1–3 (Fig. 1B). Locations 1, 5, 6 belong to quarry 1, locations 2–4 to quarry 2, location 7 to quarry 3, and these locations are 130 base of several trunks 2,100 m apart from each other (Fig. 1B). Outcrops at three Longshan quarries mainly present Guanshan Member of Wutong Formation, which is widespread in the lower reaches of the Yangtze River of China. The age of Guanshan Member is Famennian, according to the spore assemblage attributed to the *Aneurospora asthenolabrataitalicRadiizonates longtanensis* zone (14). At Longshan quarries, the Guanshan Member is characterized by quartzose sandstone with interbedded mudstone and siltstone. The stratigraphic column (SI-1 Fig. S1) is the same as illustrated in previous works (13, 15). The *in situ* plants in locations 1–6 were found from the third, first, third, fifth, fourth, fourth beds, respectively (SI-1 Fig. S1).

**Fig. 3.**
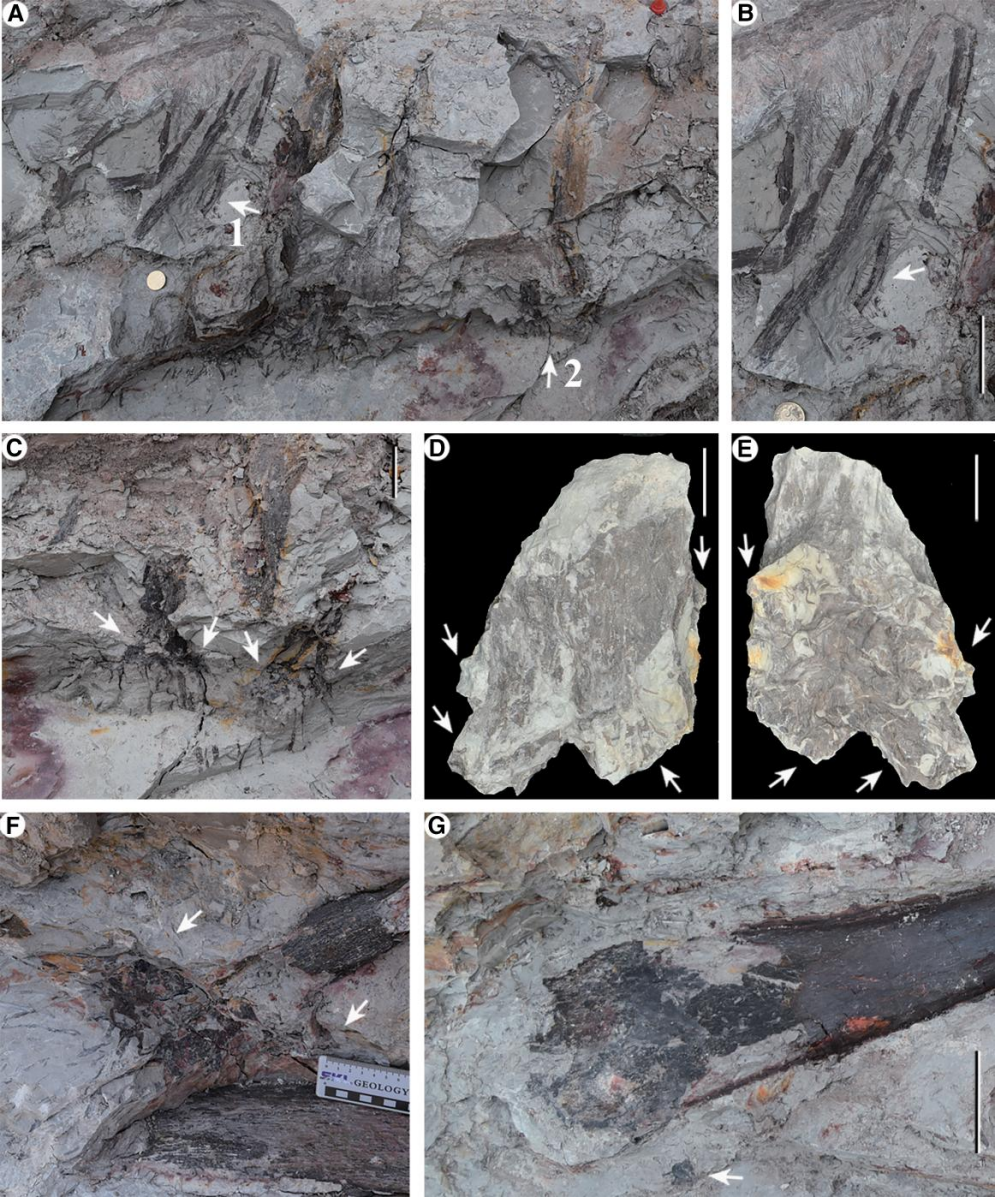
*In situ* trunks with/or rooting systems from level 2 of location 1 (excavated in October 2017). A) Lateral view of part of Location 1, arrow 1, 2 enlarged in (B, C). B) Enlargement of arrow 1 in (A), five trunks of junior plants, arrow indicating the thinnest trunk in this forest. C) Enlargement of arrow 2 in (A), two trunks of junior plants, white arrows indicating short lobes of rhizomorphs. D, E) Two opposite sides of the trunk and root of a junior plant (diameter of trunk ≤ 3 cm) in SI-1 Fig. S4C (black arrow). Arrows indicating four lobes of the rhizomorph. PKUB17402. F) A trunk, white arrows indicating lobes of the rhizomorph. Same plant as in SI-1 Fig. S8D. G) The thickest trunk, arrow indicating possible lobe's impression of rhizomorph. Same plant as in SI-1 Fig. S8E. Scale bars = 1 cm (D, E), 2 cm (coin, C), 5 cm (B), and 10 cm (F).

**Fig. 4.**
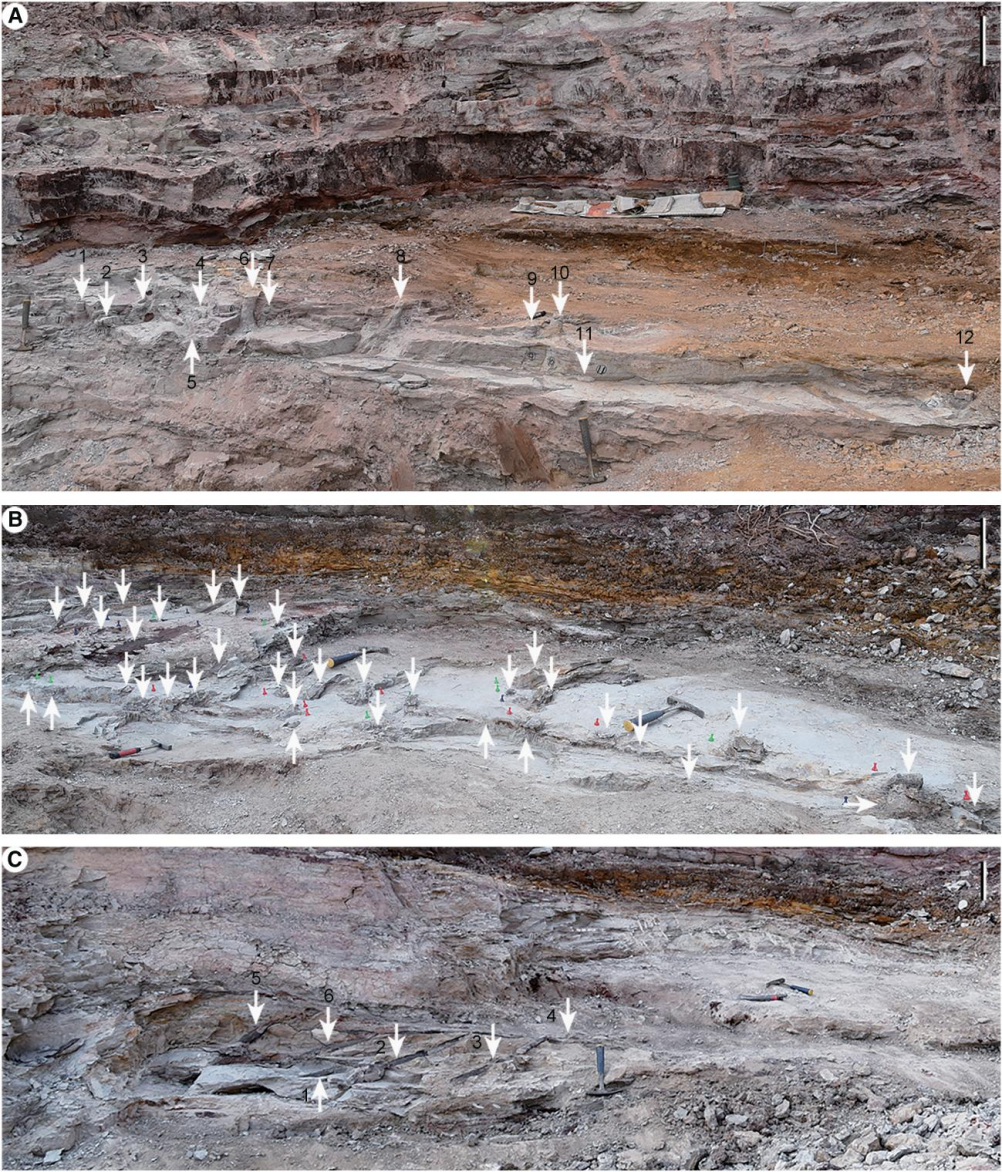
Oblique top view (from east to west) of numerous *in situ* lycopsid (*Heliodendron*) trunks and/or rooting systems (arrows) in level 1 (A, excavated in May 2017), level 2 (B, excavated in October 2017) and level 3 (C, excavated in December 2017) of location 1. A) Arrows 1–3, 6–8, and 9, 10 indicating parts excavated and enlarged in SI-1 Figs. S3A, B–G, H–I, respectively. B) *In situ* lycopsids (arrows) from level 2, successively excavated in June (as enlarged in SI-1 Fig. S4), October (SI-1 Figs. S5 and S6) and December (SI-1 Fig. S7) of 2017. C) Arrows 1–6 indicating stems as in SI-1 Fig. S8A, B (arrows 1–6), arrows 5, 6 indicating two trunks excavated and enlarged in SI-1 Fig. S8C, D (black arrows 1, 2), respectively, arrow 2 indicating a trunk enlarged in SI-1 Fig. S8G. Scale bars = 20 cm.

**Fig. 5.**
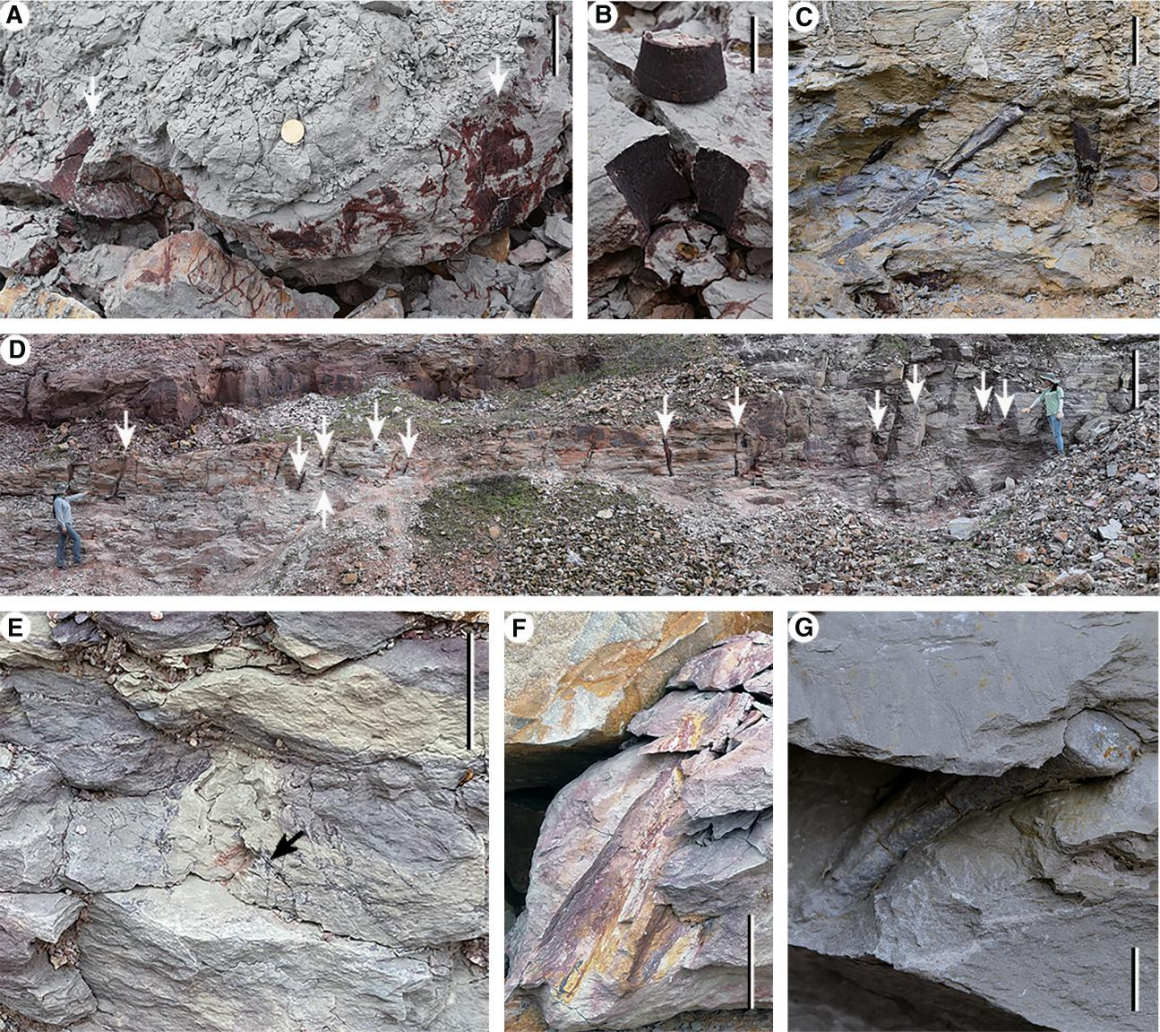
*In situ* lycopsid trunks from locations 2 (A, B), 3 (C), 4 (D), and two in situ trunks and a rooting system of probable lycopsid affinity from locations 5 (E), 6 (F), and 7 (G). A) Fallen block from highwall in SI-1 Fig. S14A, arrows indicating stems as in SI-1 Fig. S14E. B) A stem in fallen block from highwall, same as in SI-1 Fig. S14B. C) Stems from highwall, with the details in SI-1 Fig. S15. D) Stems (arrows) from highwall, same as in SI-1 Fig. S16B, with the details in SI-1 Fig. S16. E) A root from highwall, black arrow indicating root. F) A stem from fallen block. G) A stem from fallen block. Scale bars = 1 cm (G), 5 cm (A, B, C, E, F), 1 m (D).

**Fig. 6.**
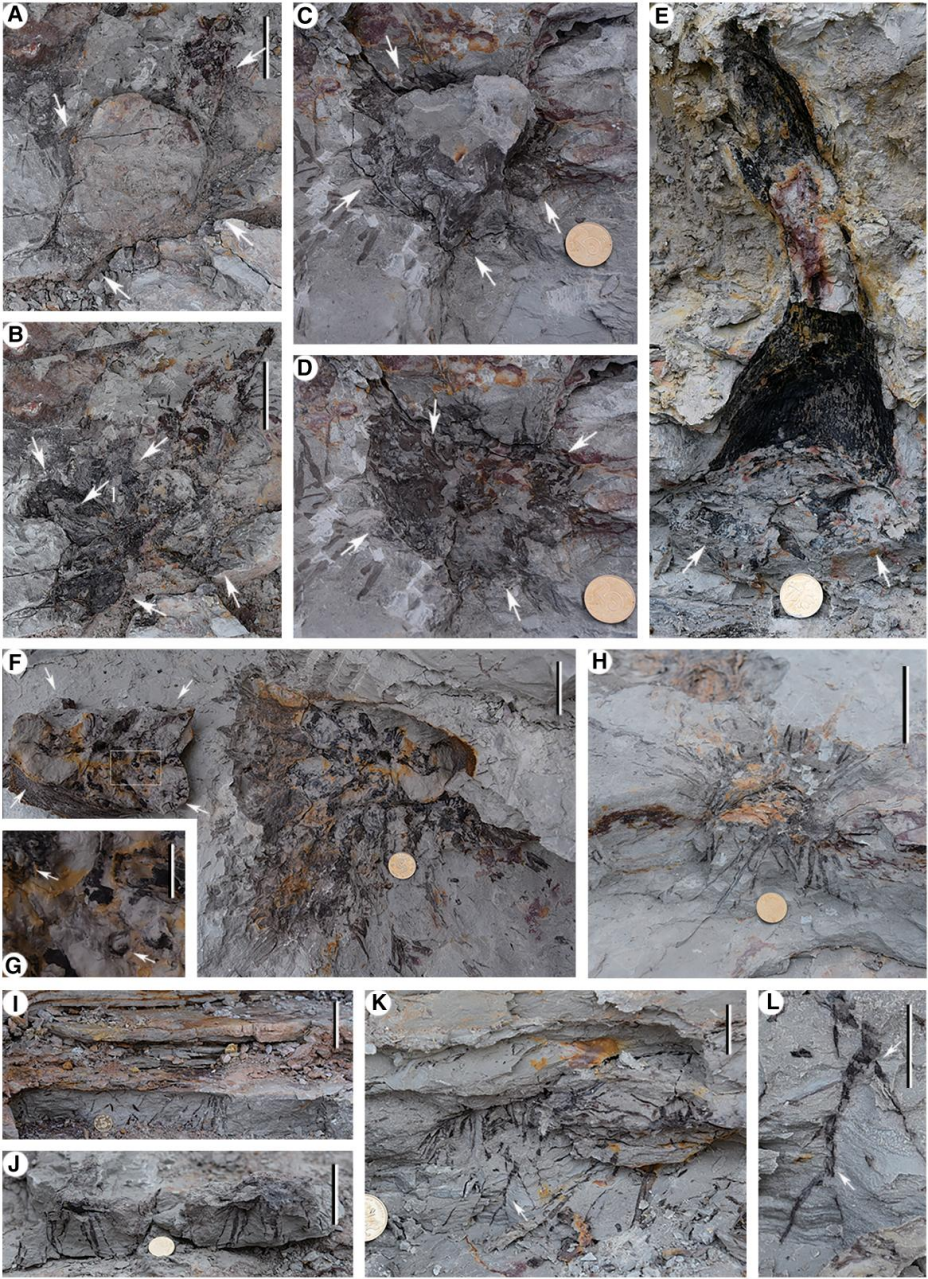
Trunks with rhizomorph bearing four short lobes (arrows in A–F, except arrow 1 in B) and roots or root scars from location 1. A, C) Two trunks with rooting system from levels 1, 2, respectively. (C) Enlarged in SI-2 Fig. S2A. B) Excavation of the rooting system in A, arrow 1 indicating rootlet scar. D) Excavation of the rooting system in (C), and picture enlarged in SI-2 Fig. S2B. E) A trunk with expanded base and rooting system from level 3. F) Cast and mould of the trunk, same as in SI-1 Fig. S7G–I. A rooting system with short rhizomorph lobes (arrows) bearing roots and root scars. G) Enlargement of part in (F) (rectangle), showing two root scars (arrows). H) Rhizomorph with radially arranged roots, same as in SI-1 Fig. S6H, after excavation. I–L) Roots on highwall, L representing enlargement of part in K (arrow), showing bifurcated root. Arrows indicating bifurcation points. Scale bars = 5 mm (G), 1 cm (L), 2 cm (coin diameter, K), and 5 cm (A, B, F, H–J).

Local companies have been quarrying Guanshan Member sandstone at Longshan Mine for many years. Numerous *in situ* lycopsids (locations 1–4) have been observed through more than 10 surveys in recent years (Figs. 2–4, 5A–D, 6, SI-1 Figs. S2–S12, and S14–S16), possible Crinoidea fossils (location 1, SI-1 Fig. S13), and probable lycopsids (locations 5–7, Fig. 5E–G) were found in quarry highwalls or in fallen blocks from highwalls. In 2017, an excavator was hired to build a ca. 15 m^2^ quadrat in location 1 (GPS: N 30°52′01.15″, E 119°44′21.38″) and the quadrat was then manually excavated layer by layer (Figs. 2–4, 6, and SI-1 Figs. S2–S13). The vertical stems in location 2 were preserved in fallen blocks of gray mudstone (Figs. 5A, B and SI-1 Fig. S14). Several oblique or vertical stems occur in yellow mudstone along the highwall in location 3 (Figs. 5C and SI-1 Fig. S15). The layer of vertical stems in location 4 is ca. 2 m thick (Figs. 5D and SI-1 Fig. S16A, B), in which at least 12 stems stand in gray siltstone along the highwall (SI-1 Figs. S16C–I). A root was found in purple yellow mudstone along the highwall in location 5 (Fig. 5E). From fallen blocks, two oblique stems were found in purple gray mudstone at location 6 (Fig. 5F) and gray mudstone in location 7 (Fig. 5G), respectively.

In the quadrat at location 1, the trunks or rooting systems of *in situ* lycopsids were preserved in ca. 1 m thick gray mudstone between sandstone; three fossil-root bearing levels are 100, 158, and 192 cm above the sandstone base, respectively (Fig. 1C, left). Within these three levels, there are several thin intercalations of siltstone and sandstone. In 2021, after rock collapse, several *in situ* lycopsid trunks and roots were discovered 10 m south of the quadrat (SI-1 Figs. S2A, black arrow, and S9). Some lycopsid stems or branches were exposed obliquely or horizontally on the bedding plane (Figs. 2I, 4B, C and SI-1 Fig. S11). Except from the lycopsid, possible Crinoidea fossils (SI-1 Fig. S13) were found at the fossil-bearing levels, while some fern-like plants appeared above (Fig. 1C, left). Seed plant *Latisemenia longshania*, progymnosperm *Archaeopteris* sp., leaf cushions of *Leptophloeum rhombicum*, reproductive organs of lycopsids and an undetermined sphenopsid were previously described in quarry 2 of Longshan (13, 15).

### Systematics

**Class** Lycopsida

**Order** Isoёtales sensu lato

**Suborder** Dichostrobiles (16)

**Family** Incertae sedis

*Heliodendron longshanense* gen. et sp. nov. Qin et al.

**Etymology:** generic name from Greek “Helio-” (sun) and “-dendron” (tree), referring to radially arranged rhizomorph lobes; specific epithet indicating the locality (Longshan sections) where the fossil plant was collected.

### Holotype designated here

PKUB17401 (The Department of Geology, Peking University, Beijing, China) (Fig. 2B and C).

### Paratypes

PKUB17402 (Figs. 3D, E and SI-1 S4C, black arrow), PKUB17403 (SI-1 Fig. S9C, right part), PKUB17404 (SI-1 Fig. S4D, near the white arrow), PKUB17405 (SI-1 Fig. S10C and D), PKUB17406 (SI-1 Fig. S11C), PKUB17407 (SI-1 Fig. S12C), PKUB17408 (SI-1 Fig. S12E), PKUB17409 (SI-1 Fig. S12G), and PKUB17410 (SI-1 Fig. S12J).

### Type locality

Near the Shiying village, Lincheng Town, Changxing County, Zhejiang Province, China.

### Horizon and age

Guanshan Member of Wutong Formation, Upper Devonian (Famennian).

**Diagnosis:** Small tree-like lycopsid with four short-lobed cormose rhizomorph. Each lobe divided once or rarely twice. Roots arranged radially and 1–2 times dicotomized. Root scars almost circular. Stem distally divided several times. Leaf cushions and bases narrow fusiform and helically arranged. Vegetative microphylls in linear shape.

The rooting systems and vertical trunks of *in situ* lycopsid *Heliodendron longshanense* occur in three levels at/near the quadrat of location 1 (Figs. 1C, 2–4, 6, SI-1 Figs. S2–S10, and S12A–D, F), some *in situ* trunks (Figs. 5A-D, F, G and SI-1 Figs. S14–S16) and a rooting system (Fig. 5E) in other six locations. The rooting systems of *Heliodendron longshanense* are usually connected to trunks. There are about 40 trunks in level 1 (Figs. 1C, 4A, 2B-C, F-H, 6A, B, SI-1 Figs. S3, and S9A, arrow 1, B, E, F), with the density being up to 13/m². Several of them are oriented obliquely to the southeast, northwest, or north, and the others are perpendicular to the bedding plane. More than 160 trunks are distributed in level 2 (Figs. 1C, 2A, D, J, 4B, 6C, D, F, H, SI-1 Figs. S4–S7, S9A, arrow 2, C, D, black arrow, S10, S12A-D, F), with a density being up to 42/m² (Fig. 1C, pink rectangle, enlarged in SI-2 Fig. S4). Most of them are directed towards west or northwest while few are in other directions, and the rest are vertical. Only 12 trunks were found in level 3 (Figs. 1C, 4C, 6E, SI-1 Figs. S8, and S9D, white arrows), with 8 trending to northwest and 3 vertically.

**Fig. 7.**
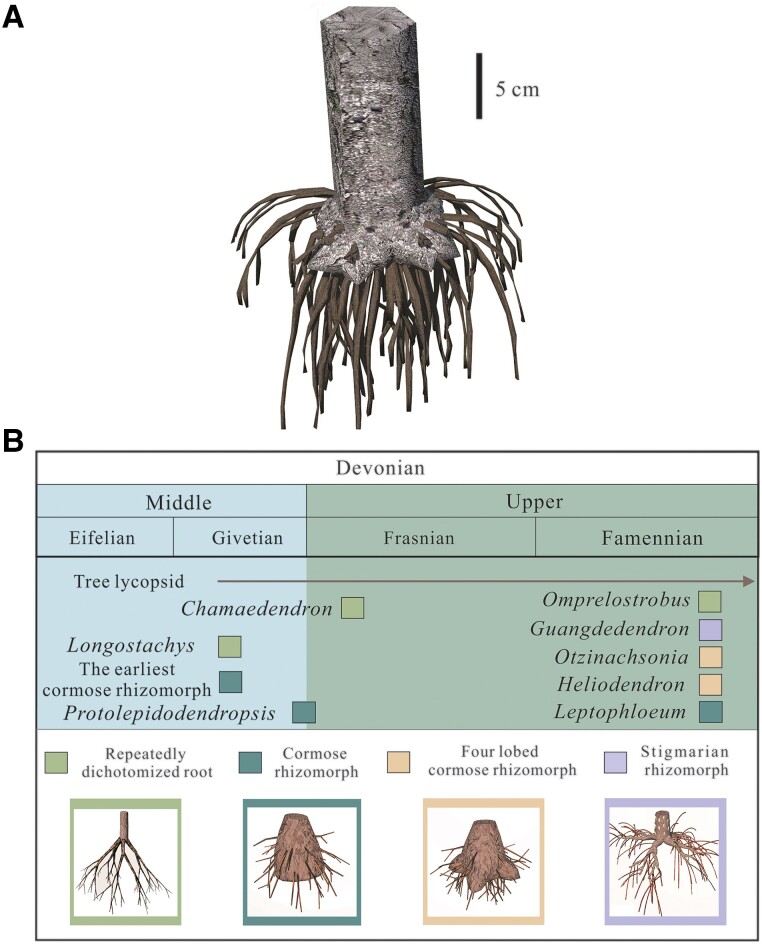
A) Reconstruction of *Heliodendron*'s rooting system. B) Rooting systems and their initial appearance time of Devonian tree lycopsids. Schematic diagram and occurrence time of four types of rooting systems in Devonian and their representative lycopsid plants. *Longostachys* (7); *Chamaedendron* (8); *Omprelostrobus* (17);? the earliest cormose rhizomorph (18); *Protolepidodendropsis* (3); *Leptophloeum* (10); *Otzinachsonia* (9); *Guangdedendron* (4, 19); *Heliodendron* (this paper).

The rooting systems are 4.6–13.5 cm deep and each rhizomorph has four short lobes, which extend obliquely downwards or horizontally (Figs. 2A–H, 3C–F, 6A–F, 7A, SI-1 Figs. S4C, S5G, S6G–M, and S10F–H). The lobes are up to 5.6 cm long and unequally divided once or seldom twice (Figs. 2A, SI-1 FIgs. S5G, S6G, and S10F–H). Generally, the lobes are short and easily broken, so they are not complete when taken out, like plant in Fig. 2B and C, the rhizomorph is clearly divided into four unequal parts, but the ends are broken off (white arrows in Fig. 2G and H). The roots are 3.3–(11.9)–23 cm long and 1.2–(2.2)–4.8 mm in diameter and are radially arranged on the rhizomorph (Figs. 2A, H, 3C, F, 6A–F, H–L, SI-1 Figs. S3C, I, S4B–E, S5G, S6G, H, M, S7G, H, S8C, D, S9C, and S10F–H), and sometimes dichotomize once or twice (arrows in Figs. 6K, L, SI-1 Figs. S3D, and S4B, D, E). Root scars are almost circular and 2.1–(3.0)–4.8 mm in diameter (Figs. 6B, arrow 1, F, G, SI-1 Figs. S7I, S9C, and S10G, arrow).

The diameter of over 210 *in situ* trunks ranges 0.7–(5.3)–21 cm, of which 95% are less than 10 cm. The trunk with minimum/maximum diameter is illustrated in Figs. 3A, B (white arrow) and 3G. The remaining (obliquely) vertical trunks reach a height of 64–176.9 cm (SI-1 Figs. S8E, G, S10A, B, and S16E), and the stems on bedding plane are up to 147 cm long (Figs. 2I, SI-1 Figs. S8A, black arrow, and S11A). The upper parts of the stems may dichotomize once or twice at 30–60° to form distal branches, that are reduced by about half in diameter (Figs. 2I and SI-1 Figs. S11A, C–E). The diameter of the stem in Fig. 2I prior to branching measures 4.3 cm, with the two resulting branches measuring 2.4 and 2.6 cm, respectively.

**Fig. 8.**
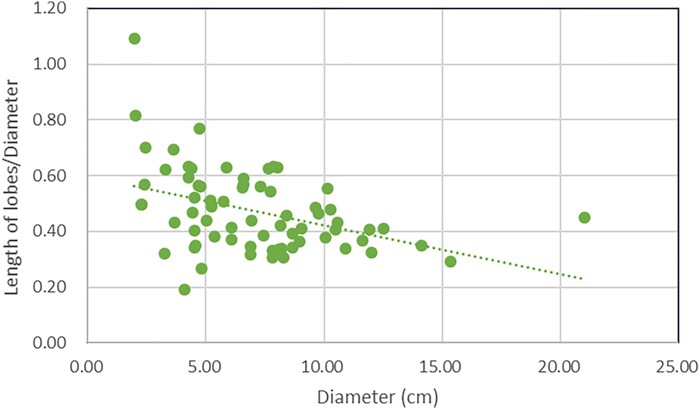
The trend of the ratio of lobes’ length to diameter of the *Heliodendron* as the diameter increases.

Juvenile individuals of *Heliodendron longshanense* typically grow in clusters (Fig. 3A and B), with their rhizomorph exhibiting four undivided lobes (Figs. 3D, E and SI-1 Fig. S6M). However, adult individuals exhibit rhizomorph lobes with larger length and further division (Figs. 2A, D, 6C, SI-1 Figs. S5G, S6G, and S10F–H). Sixty-nine *in situ* plants were preserved with trunk and measurable length of rhizomorph lobes. The ratio of lobes’ length to diameter decreased as trunk diameter increased (Fig. 8).

Linear vegetative leaves (Figs. 2J and SI-1 Fig. S12A–G) with entire margins are 2.7–(4.7)–6.0 cm long and 0.7–(0.8)–1.1 mm wide, and sometimes remained on the base of trunks (Figs. 2J, 3A, B and SI-1 Fig. S12A–D, F, G). Narrow fusiform leaf cushions (SI-1 Figs. S9F, S10A, B, S14B–I, S15F, and S16F, G) or leaf bases (SI-1 Figs. S8F, S10C, D, S11B, and S12G–J) are closely and helically arranged in parastichies on the stems and branches.

## Discussion

### Classification of the rooting system and allometry in early tree lycopsids

Devonian tree or tree-like lycopsids have a high morphological diversity in the shoot but there are only four types of rooting system. As shown in Fig. 7B, these root types include: (i) repeatedly dichotomized root, which is seen in the Middle Devonian *Longostachys* (7), Late Devonian (Frasnian) *Chamaedendron* (8), and *Omprelostrobus* (17); (ii) cormose rhizomorph with a swollen base bearing roots, which possibly originated in the Middle Devonian (18) and typifies late Givetian-Frasnian *Protolepidodendropsis* (3) and Famennian *Leptophloeum* (10); (iii) lobed cormose rhizomorph with attached roots, which is recorded in Famennian *Otzinachsonia* (9) and *Heliodendron* (this paper); (iv) *Stigmaria* rhizomorph with four branched axes bearing roots, which occur in Famennian *Guangdedendron* (4, 19).

*Otzinachsonia* (9) and *Heliodendron* share four short-lobed cormose rhizomorph. However, the rhizomorph of *Otzinachsonia* is differentiated into distinct furrows and undivided lobes, concentrated with small and large root scars, respectively. In addition, the leaf scars/bases on the trunk of *Otzinachsonia* are horizontally elliptical or triangular, while those of *Heliodendron* are vertically fusiform. As a small tree lycopsid, the Early Carbonierous (Mississippian) *Protostigmaria eggertiana* possesses a lobed cormose root (20). It differs from *Heliodendron* by having 2–13 rhizomorph lobes and round leaf scars/bases arranged in low helices or pseudowhorls on the stem.

*Guangdedendron* forming Xinhang lycopsid forest was found in the Leigutai Member of the Wutong Formation (4), occurring a little later than *Heliodendron* in the Guanshan Member. The linear distance between Xinhang and Lincheng forests is about 25 km. *Heliodendron* and *Guangdedendron* share similar stem width, and according to the calculation formula between the diameter value (*D*) and the height (*H*) (*H* = 37.5 × (*D*/2)^2/3^) (21), they are separately 0.8–8.4 m and 1.1–7.7 m high. Both of the two tree lycopsids possess dichotomously branched stem, linear vegetative leaves with entire margin, and fusiform leaf bases helically arranged in parastichies. In contrast, *Guangdedendron* is characterized by *Stigmaria*-type rhizomorph with four divided axes reaching 25.3 cm length (4) or 31 cm length (19). *Heliodendron* is characterized by lobed cormose rhizomorph with four divided unequal lobes just reaching 5.6 cm.

The rhizomorph of *Heliodendron* consists of uneven lobes. As the trunk diameter increases evidently, the length of rhizomorphic lobes increase in a less proportion, suggesting allometry. It is proposed that in the life cycle of tree lycopsids, the rhizomorph establishment precedes the elongation of stem (22). The lower growth rate in the length of rhizomorphic lobes may indicate a post-growth stage and thus correspond to the above-mentioned developmental pattern. The reduced growth rate of rhizomorph may result in inadequate mechanical support for the above-ground portion of the trees, potentially leading to collective tilt, which is a rare phenomenon in Xinhang Forest.

Many leaves of tree lycopsids abscise, forming distinct “leaf scars”, like *Chamaedendron* (8) and *Omprelostrobus* (17), However, *Heliodendron* displays leaves persisting at the base of several trunks (Figs. 2J and SI-1 Fig. S12A–D, F), suggesting that some individuals may exhibit a slower leaf shedding process or retain leaves throughout their lifespan.

### Composition of early forests

The earliest forests in the Devonian were discovered in recent years and consist of fern-like plants, progymnosperms, and/or lycopsids (1–4, 23). The exposed area of nonlycopsid fossil forests (Middle Devonian Gilboa and Cairo forests from New York of United States) range from 1,200 to 3,000 m^2^ and the density of large trees is less than 1/m^2^ (1, 2). The Late Devonian Svalbard forest from Norway and Xinhang forest from China are (mainly) composed of small lycopsid trees (3, 4). The area of Svalbard forest is unclear and the average of tree density is 14/m^2^. The exposed area of Xinhang forest is at least 250,000 m^2^, and it is the largest Devonian fossil forest and even larger than many Carboniferous fossil forests; the local density of trees is up to 38/m^2^.

The Guanshan Member is ca. 75 m thick at the Longshan section (13, 15) (SI-1 Fig. S1). In the mid-lower part of Guanshan Member of this section, the *in situ* plants from locations 1–7 occur in mudstone or siltstone as thin beds or wide lens between sandstones (Figs. 1, 2, 4–6, SI-1 Figs. S1–S12, and S14–S16), although the plant-bearing horizons could not be accurately correlated among the seven locations due to long distance and the covering Quaternary deposits. Within an area of 75 hectares limiting these seven locations, *in situ* lycopsids are distributed as small patches. The density of *Heliodendron* trees is up to 42/m^2^ in Lincheng forest (Fig. 1C, pink square), indicating that dense growth may be normal for Devonian tree lycopsids. Corresponding with such a growth pattern, these trees have a long stem producing a simple crown containing rare dichotomous terminal branches to prevent shading among individuals (Figs. 2L, SI-1 Figs. S8E, G, S10A, B, and S11).

The Middle Devonian Gilboa forest mainly consist of *in situ* aneurophytes and cladoxylopsids, together with a few heterochthonously preserved lycopsids; while Cairo forests consist of *in situ Archaeopteris*, cladoxylopsids, and few possible lycopsids (1, 2). Late Devonian (Frasnian) Svalbard forest was made up of small lycopsid trees, with *in situ* archaeopteridalean trunks recognized (3). The Late Devonian (Famennian) Xinhang forest is characterized by small lycopsid trees and there are also other plants, such as *Xinhangia* and *Sublepidodendron* (4, 24, 25). In the Lincheng forest, most *in situ* trees are recognized as lycopsids (locations 1–4), with distinguishable leaf bases or cushions. The trees with rooting systems are identified as *Heliodendron longshanense* in location 1, while those along the highwalls or in fallen blocks of locations 2–4 are also lycopsids, and the two trunks and a rooting system in locations 5–7 may belong to lycopsid. Carboniferous and Early Permian swampy forests are complicated in vertical stratification, with both canopy-forming trees and understory plants of multiple affinities including lycopsids, horsetails, ferns, and gymnosperms (26–28). Therefore, the Late Devonian forests with small lycopsid trees may represent a transition from the Middle Devonian forests majorly consisting of cladoxylopsids and archaeopterids, to later forests usually dominated by giant lycopsid trees.

### Environment and preservation of tree lycopsids

The Upper Devonian Wutong Formation, widespread in the Lower Yangtze Valley of China (including Zhejiang Province), consists of the underlying Guanshan Member mainly with thick quartz sandstone and the overlying Leigutai Member with interbedded mudstone and quartz sandstone (29). The stratigraphic, sedimentary, and geochemical analyses concluded that the Wutong Formation represents a coastal (littoral) environment near the palaeoequator (4, 30–33). The lithology and sedimentation of the Wutong Formation indicate that a general sea level regression occurred from Guanshan Member to Leigutai Member (31, 32, 34). The *in situ* plants in Guanshan Member were more easily disturbed by sea flow than those in Leigutai Member. Possible Crinoidea fossils may suggest that Lincheng forest was influenced by the sea water.

In the Carboniferous, giant tree lycopsids with extensive stigmarian roots dominated coal swampy habitats, while the smaller tree lycopsids with lobed cormose bases reflected nonswampy conditions (5, 20). *Guangdedendron* and *Heliodendron* indicate that the Late Devonian tree lycopsids with both types of rooting system lived on clastic substrates. Many *in situ* trunks or stems in location 1 (Figs. 1C, 4B, C, SI-1 Figs. S4A–D, S5, S6A–F, L, and S7A, B, arrows 1, 3, E, G, S8, S9D–F, S10A–D, S12A–D, F), 3 (Figs. 5C and SI-1 Fig. S15A, C–F), and 4 (Figs. 5D and SI-1 Fig. S16B) were directed obliquely to the northwest or west. This probably indicates the main direction of sea water flow when the plants were buried. The four lobed rhizomorphs of *Heliodendron* bore numerous roots often extended obliquely downwards, providing mechanical support to the plant (Figs. 2A–H, 6, SI-1 Figs. S3C, D, I, S4B–E, S5G, S6G–M, S7G–I, S8C, D, S9C, and S10F–H). The roots are shallow penetrating, moderately dichotomous and radially arranged (e.g. Figs. 2A, 6F–L, SI-1 Figs. S5G, and S6G, H, M), thus reflecting poor drainage and a high-water table.

The *in situ* lycopsid trunk casts crossing the sedimentary beds of the Upper Devonian Wutong Formation have been reported from several localities in South China (4, 17), and they resulted from burial events, i.e. strong flood carrying large amount of sediments. Each layer of *in situ* trunks may correspond to a separate flood event, during which rapid water flow tilted the trunks and buried them with leaves in abundant sediment. The exposed portions of the tree lycopsids above the sediment were later damaged during subsequent floods or decay, resulting in the truncated tops of the preserved trunks. Tree lycopsids located on the periphery of the flood disturbance zone may be less affected, potentially allowing them to propagate and recolonize the habitat. This process may be repeated several times, resulting in the preservation of multiple layers of *in situ* fossil forests within the strata (3). However, the different fossil-bearing horizons may represent a single, long-lived community that fluctuated in size and distribution due to the influence of seawater.

## Supplementary Material

pgae241_Supplementary_Data

## Data Availability

All data are included in the manuscript and/or supporting information.
