# Supplementary material for: In situ forest with lycopsid trees bearing lobed rhizomorphs from the Upper Devonian of Lincheng, China
Source: PNAS Nexus. 2024 Jun 15;3(7):pgae241. doi: 10.1093/pnasnexus/pgae241 (PMC11231945; doi:10.1093/pnasnexus/pgae241)
Supplement: pgae241_Supplementary_Data [file pgae241_supplementary_data.docx]

**Supplementary Information (SI)**

**SI-1**


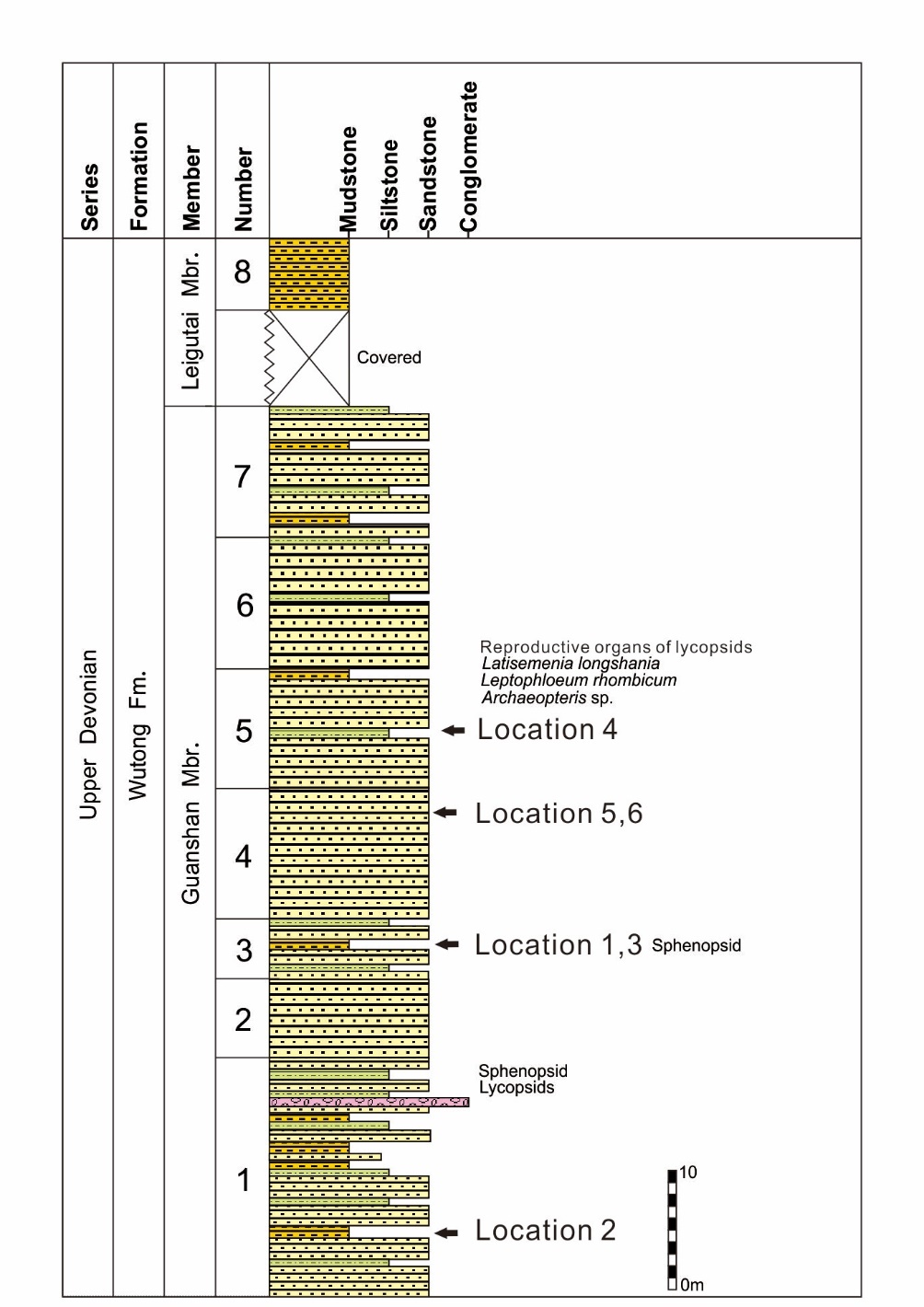


**Fig. S1.** Stratigraphic column at Longshan section (Quarry 2), Changxing County (Zhejiang, China), showing the lithology and sequence of strata, and the beds bearing plants (modified from Fig. S3 *<13>*)


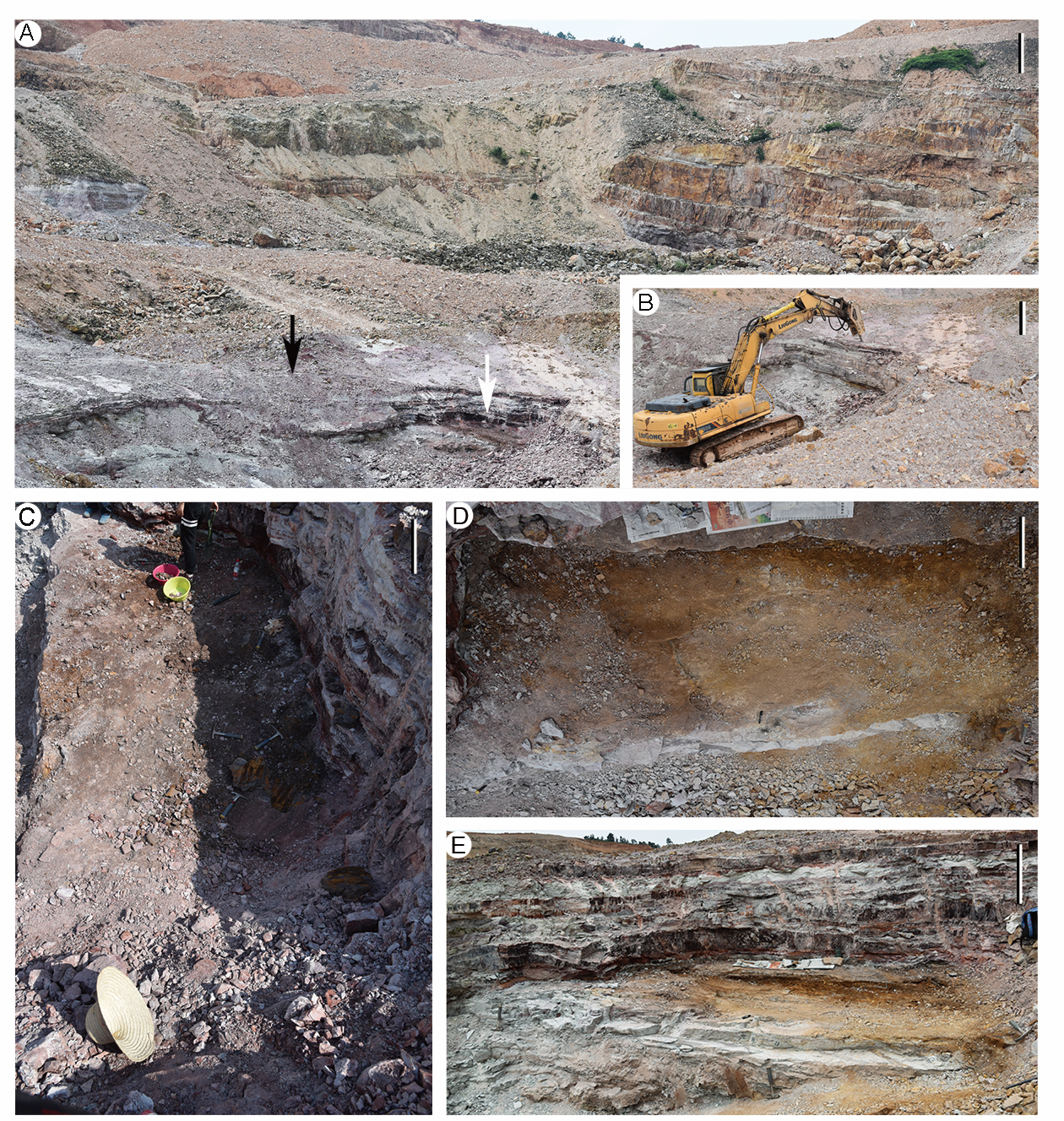


**Fig. S2.** Outcrop of location 1 in Quarry 1, before extensive excavation.

**A.** Distant view of the quadrat (white arrow), with black arrow indicating a new highwall after excavation (as in Fig. 1D, SI-1 Fig. S9).

**B.** Working of an excavator to remove overlying sandstones at site.

**C**-**E.** Top (C, D) and oblique top (E) view of location 1 from north, west and east directions, respectively.

Scale bars =27 cm (hammer length), 50 cm (C-E), 2 m (A, B).

**
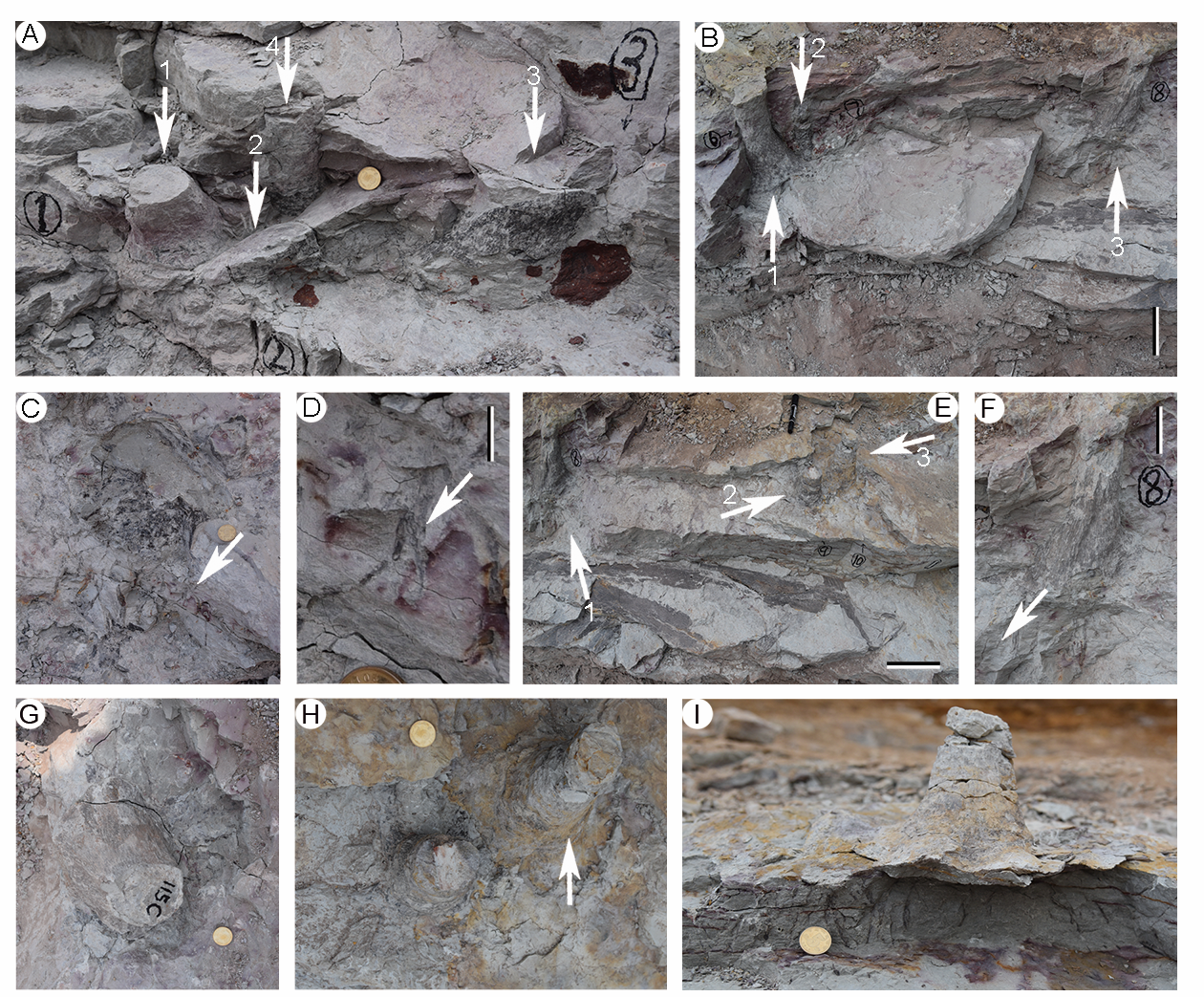
**

**Fig. S3.** *In situ* trunks with/or rooting systems from level 1 of location 1 (Fig. 4A, arrows 1-3, 6-10, excavated in May 2017). Rooting systems with four rhizomorph lobes bearing roots.

**A.** Oblique top view of four trunks (arrows), with three trunks (arrows 1-3) as those in Fig. 4A (arrows 1-3), and the fourth trunk exposed (arrow 4) after excavation.

**B.** Oblique top view of three trunks (arrows), as those in Fig. 4A (arrows 6-8), arrow 1 indicating trunk shown in Figs. 2B, C.

**C.** Bottom mould with roots after removing the trunk in B (arrow 1) and Figs. 2B, C, I-K. White arrow indicating bifurcated root.

**D.** Enlargement of arrowed part in C. Arrow indicating bifurcated root.

**E.** Oblique top view of three trunks (arrows), as those in Fig. 4A (arrows 8-10).

**F.** Enlargement of trunk in B (arrow 3) and E (arrow 1).

**G.** Top view of trunk in F after excavation.

**H.** Top view of two trunks in E (arrows 2, 3), arrow showing the same trunk in E (arrow 3).

**I.** Lateral view of excavated trunk in E (arrow 3) and H (arrow), also showing roots.

Scale bars = 1 cm (D), 2 cm (coin diameter), 5 cm (F), 10 cm (B, E).


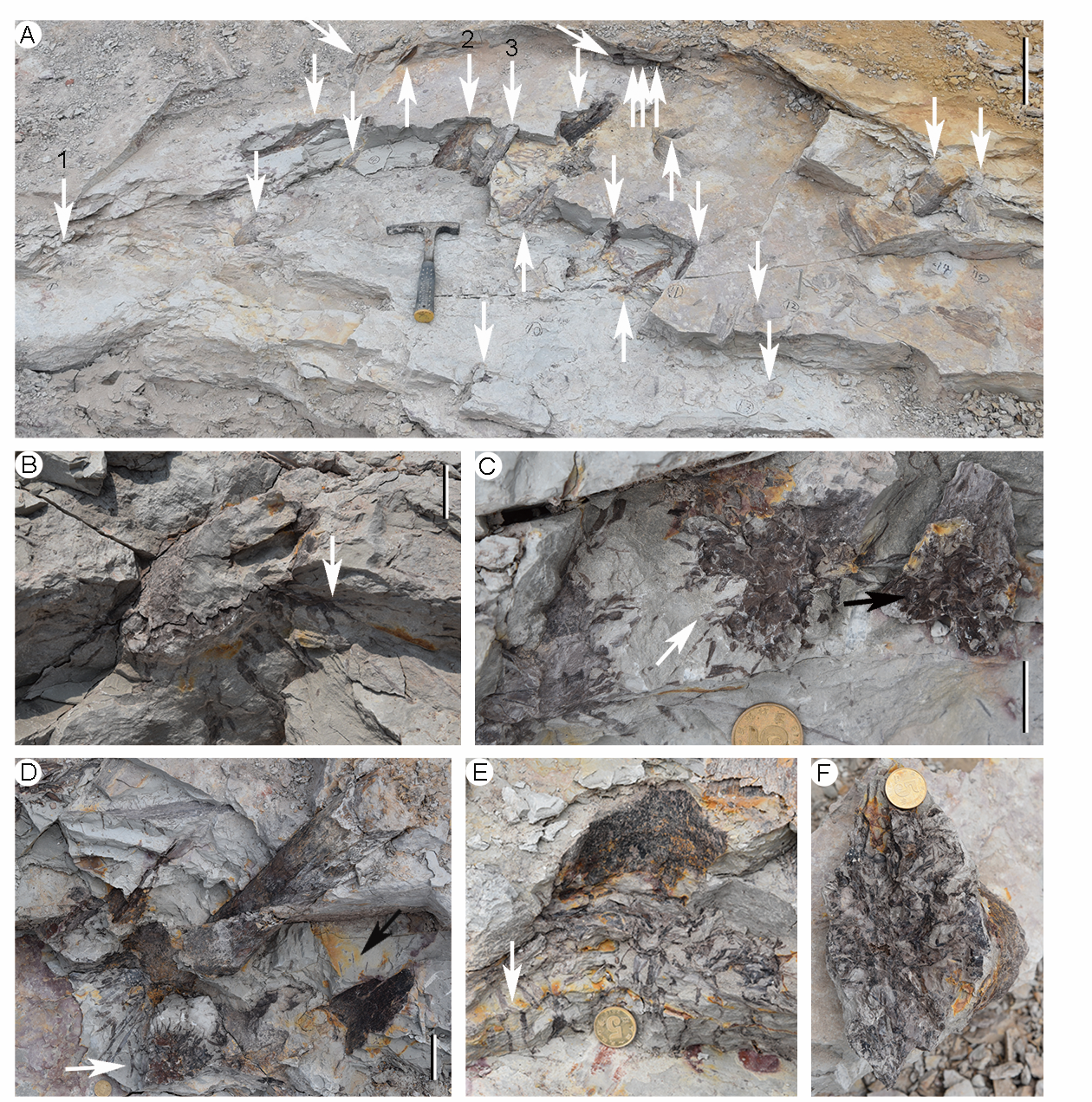


**Fig. S4.** *In situ* trunks with/or rooting systems from level 2 of location 1 (excavated in June 2017).

**A.** Multiple trunks with/or rooting systems (arrows).

**B.** Trunk with rooting system in A (arrow 1) after excavation, arrow indicating bifurcated root.

**C.** Cast of a trunk (black arrow) in the right rear of the trunk in B after excavation, showing four short rhizomorph lobes as in Figs. 3D, E; mould of the same trunk (white arrow) and roots. PKUB17402.

**D.** Two trunks in A (arrows 2, 3) after excavation, with white and black arrows indicating bifurcated roots and the place where a trunk was cut by a horizontal movement of strata, respectively. Fossil near the white arrow, PKUB17404.

**E.** Excavation of trunk in D (black arrow), showing mould and roots. Arrow indicating bifurcated root.

**F**. Cast of the trunk in E.

Scale bars = 2 cm (coin diameter, B, C), 5 cm (D), 20 cm (A).

**
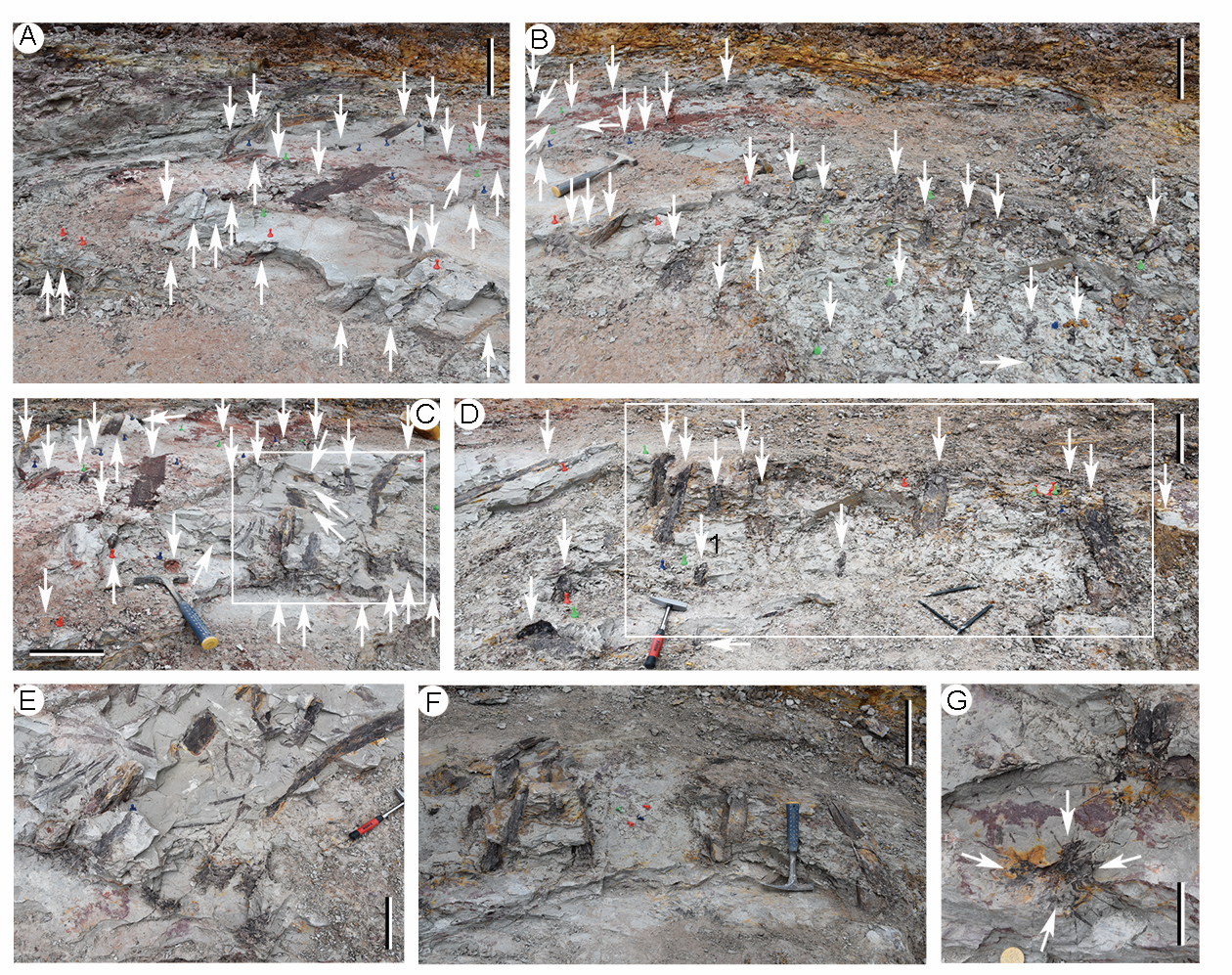
**

**Fig. S5.** I*n situ* trunks with/or rooting systems from level 2 of location 1 (excavated in October 2017).

**A, B.** Oblique top view of numerous trunks (arrows) in left and right parts of location 1, respectively.

**C, D.** Excavation of trunks (arrows) in A and B, respectively. Rectangles indicating parts enlarged in E and f after excavation, respectively.

**E, F.** Enlargement of C (rectangle) and D (rectangle) after excavation, respectively, showing trunks with/or rooting system.

**G.** Excavation of trunk in D (arrow 1) after excavation, arrows indicating four lobes of the rooting system, with the left axis dichotomizing once slightly.

Scale bars = 5 cm (G), 10 cm (D, E), 20 cm (A-C, F).

**
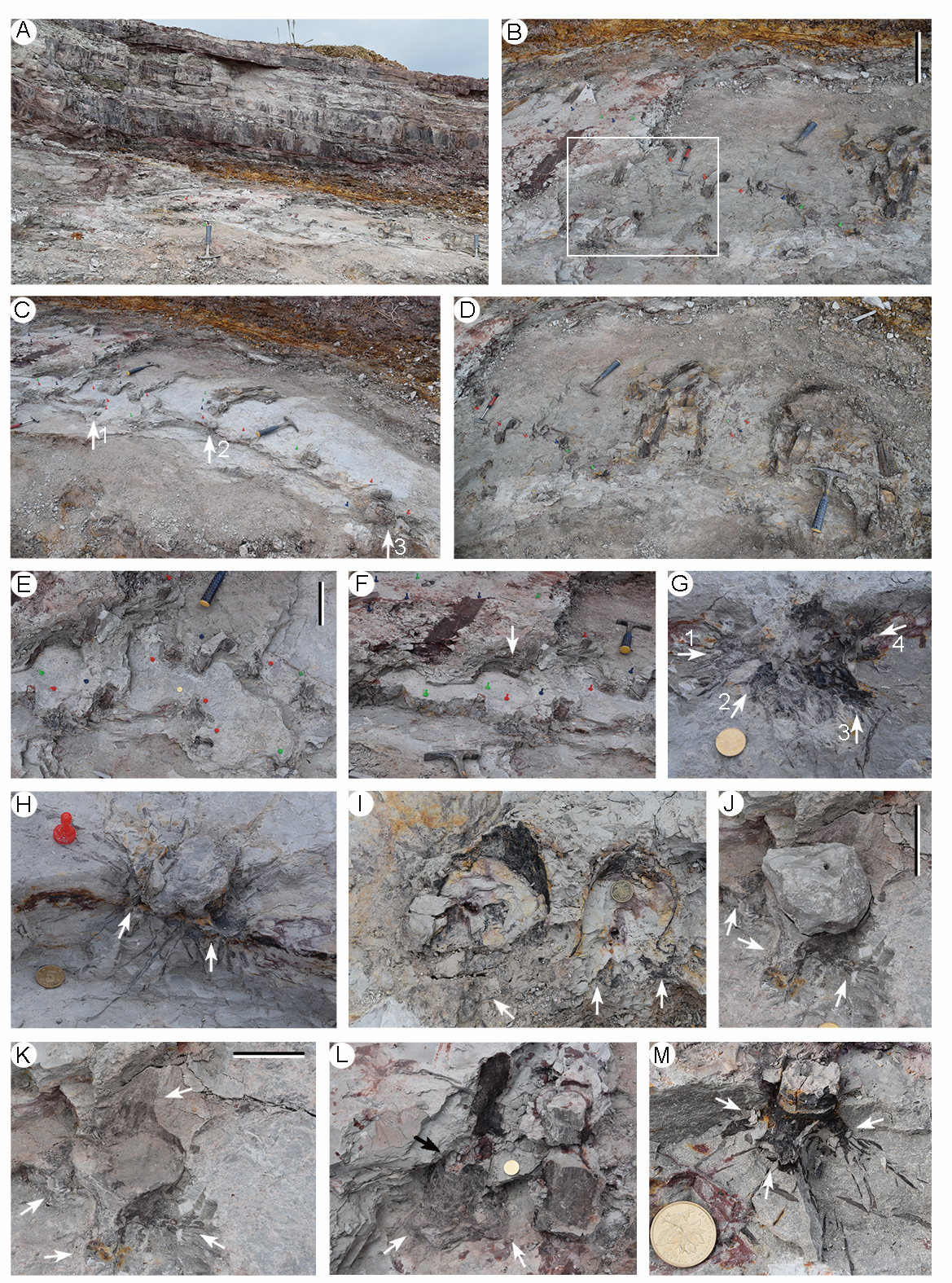
**

**Fig. S6.** Further excavation of *in situ* trunks with/or rooting systems in SI-1 Fig. S5 (excavated in October 2017).

**A.** Lateral view of location 1.

**B.** Enlargement of left part of location 1, in top view, with rectangle part excavated and then enlarged in E.

**C.** Enlargement of left part of location 1, in oblique top view. Excavation of part in B. Arrows 1-3 indicating parts enlarged in G-I, respectively.

**D.** Enlargement of right part of location 1, in oblique top view.

**E.** Excavation of part in B (rectangle).

**F.** Excavation of trunks in E. Arrow indicating part enlarged in J and K.

**G.** Enlargement of excavated part in C (arrow 1), showing rooting system with four short lobes (arrows 1-4) bearing roots, and two lobes (arrows 1, 4) dichotomizing once slightly.

**H.** Enlargement of part in C (arrow 2), showing rooting system with short lobes (arrows) bearing roots, same part as in Fig. 6H.

**I.** Enlargement of excavated part in C (arrow 3), showing two rooting systems with short lobes (white arrows) bearing roots.

**J, K.** Enlargement of successively excavated part in F (arrow), showing a rooting system with four short rhizomorph lobes (white arrows) bearing roots.

**L.** Trunks near the plant in J, K, after excavation, white arrows indicating two short rhizomorph lobes of the left plant’s rooting system (black arrow).

**M.** Rooting system with short rhizomorph lobes (white arrows) bearing roots, and enlarged in SI-2 Fig. S3.

Scale bars = 2 cm (coin diameter), 5 cm (J, K), 10 cm (E), 20 cm (B), 27 cm (the blue hammer length).

**
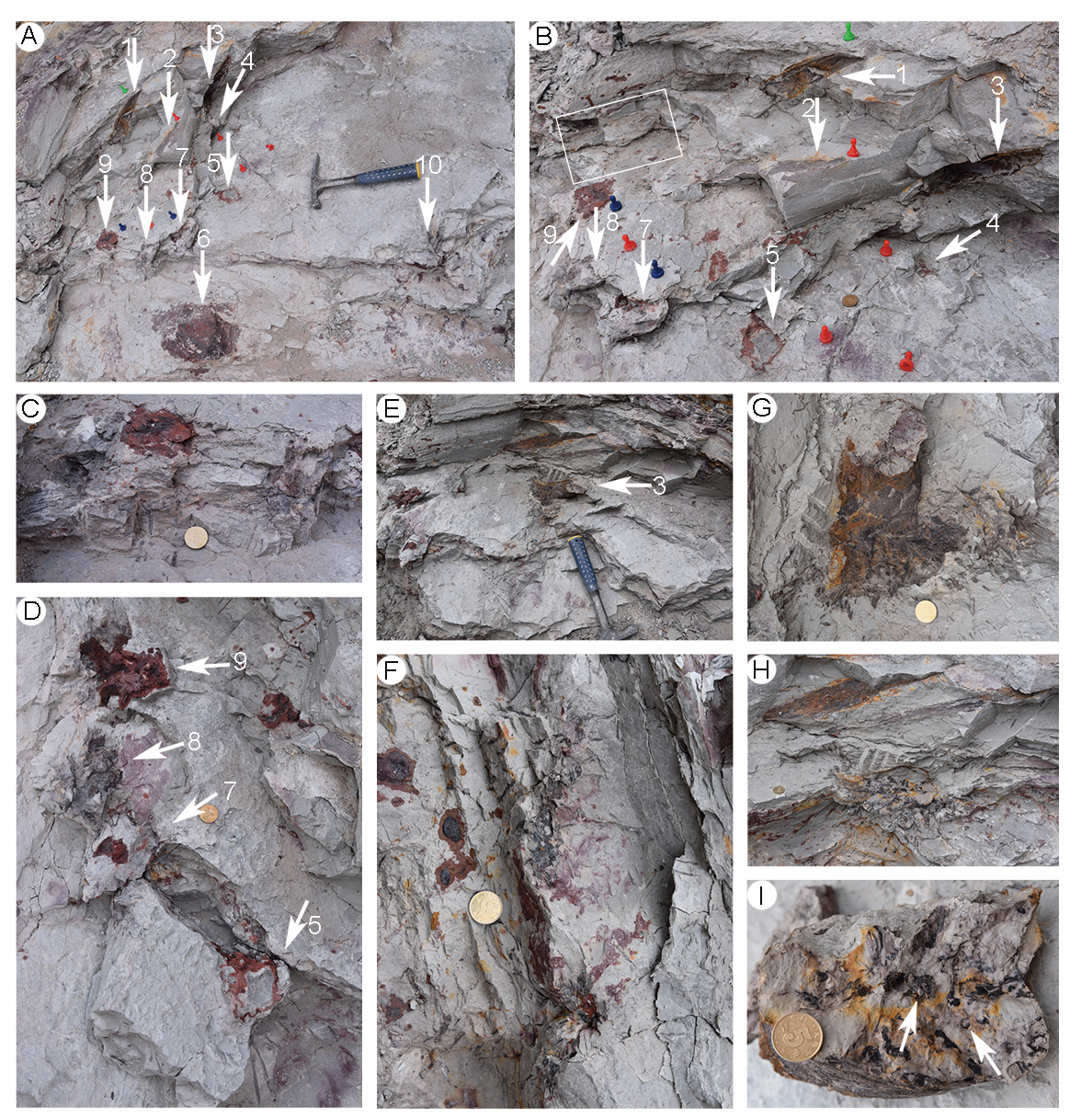
**

**Fig. S7.** *In situ* trunks (stems) with/or rooting systems from level 2 of location 1 (excavated in December 2017).

**A.** Ten trunks or stems (arrows) viewed in one direction.

**B.** Eight trunks or stems (arrows 1-5, 7-9 in a) viewed in another direction. Rectangle area excavated and enlarged in f.

**C.** Enlargement of two trunks in A and B (arrows 8, 9).

**D.** Further excavation of trunks in A and B (arrows 5, 7-9).

**E.** Further excavation of stem in A and B (arrows 3).

**F.** Further excavation of part in B (rectangle), viewed in a different direction.

**G.** Enlargement and view of arrowed trunk in E, after counterclockwise rotation.

**H, I.** Excavation of trunk in G, showing mould and cast, respectively, the same as in Fig. 6F, arrows indicating root scars.

Scale bars = 2 cm (coin diameter), 27 cm (hammer length).

**
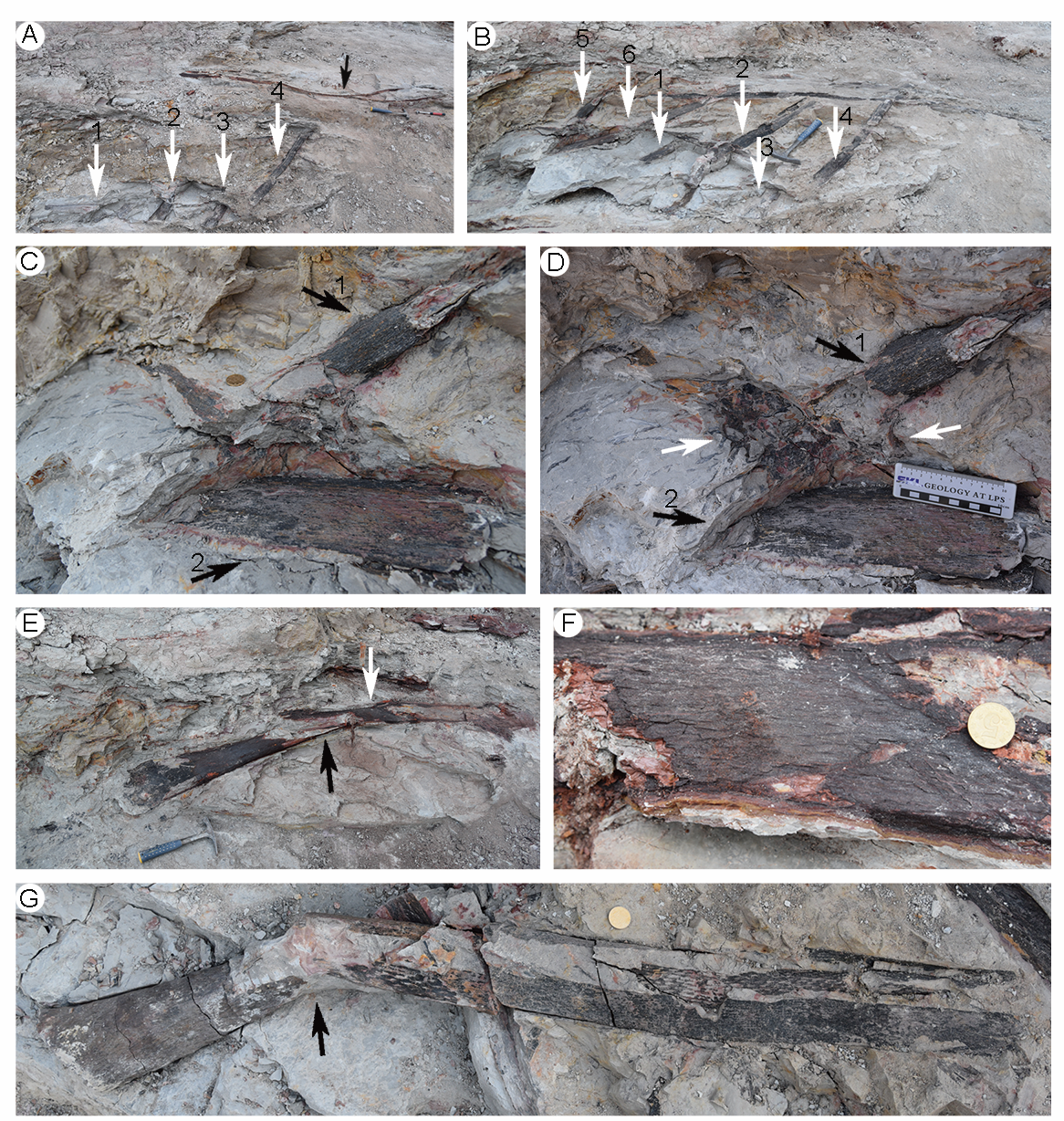
**

**Fig. S8.** *In situ* trunks with/or rooting systems from level 3 of location 1 (excavated in December 2017), same plants as in Fig. 4C (arrows).

**A.** Four vertical trunks (white arrows). Black arrow indicating a dichotomized stem along bedding plane, which is enlarged in Fig. 2I and SI-1 Fig. S11A.

**B.** Excavation of part in A to show more trunks (arrows).

**C**-**E.** Serial excavation of part in B (arrows 5, 6). Black arrows 1, 2 in C and D indicating two trunks from arrows 5, 6 in B, respectively; white arrows in D indicating short rhizomorph lobes of rooting system in B (arrow 5); white arrow in E enlarged in F. Black arrow in E indicating where the stem is broken. Trunk in D same as in Fig. 3F.

**F.** Enlargement of white arrowed part in E, showing a stem with leaf bases.

**G.** Enlargement of part in A and B (arrow 2), showing a long stem where it changes from oblique trunk to lying on bedding plane, black arrow indicating where the stem is turned.

Scale bars = 2 cm (coin diameter), 27 cm (hammer length).


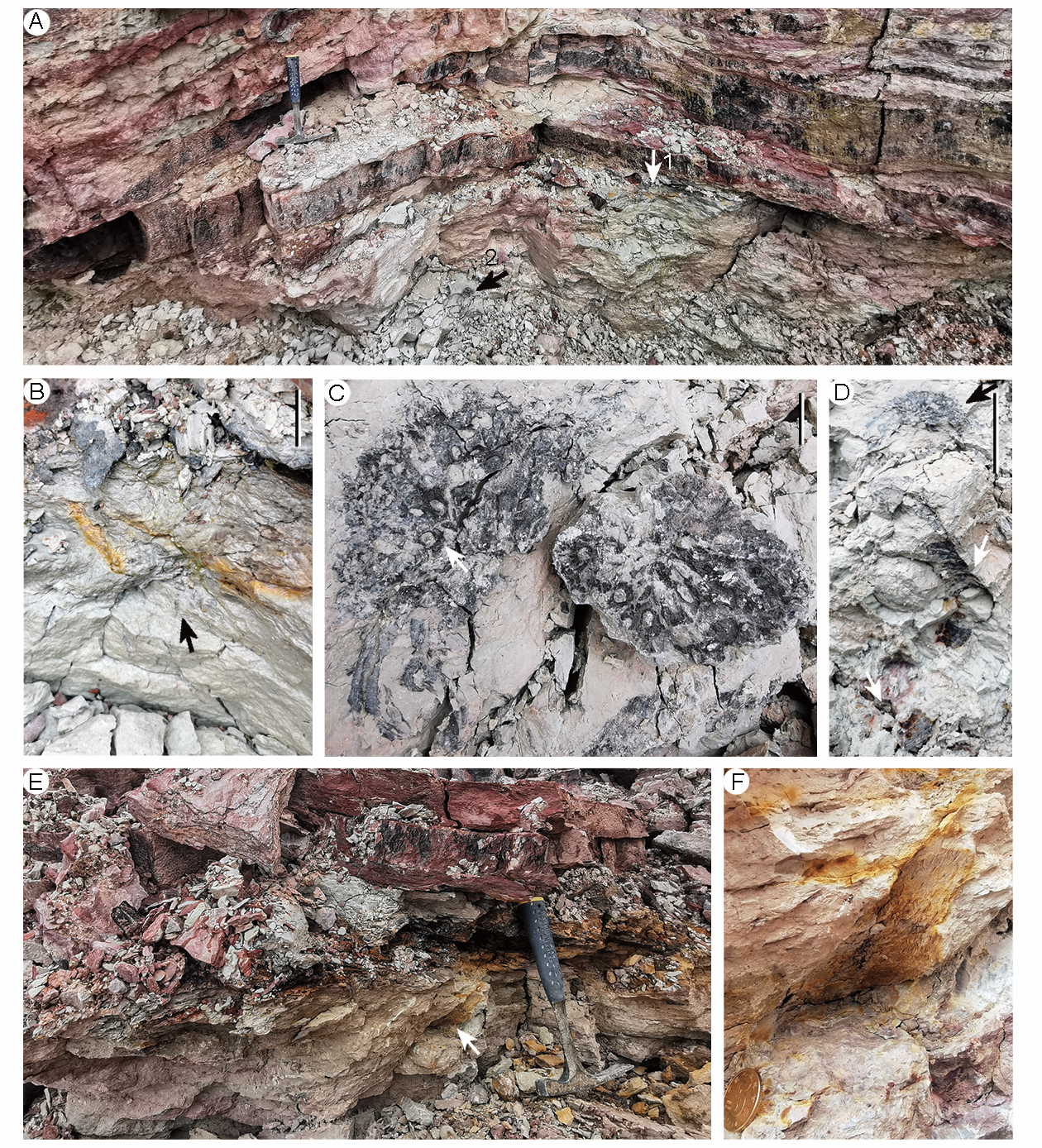


**Fig. S9.** *In situ* trunks with/or rooting systems from another highwall of location 1 (excavated in March 2021). They are about 10 meters south of the quadrat.

**A.** Highwall, with arrows 1, 2 indicating rooting systems enlarged in B and C, respectively. These two rooting systems corresponding to those in levels 1 and 2, respectively.

**B.** Enlarged rooting system with roots (arrow) from a (arrow 1).

**C.** Enlarged rooting system from A (arrow 2), with left and right parts representing mould and cast, respectively. Arrow indicating root bases. PKUB17403.

**D.** Two trunks (arrows) as in level 3 after excavation of part in C, with black arrow indicating the rooting system in C and A (arrow 2).

**E.** Highwall near that in A, arrow indicating a trunk enlarged in F.

**F.** Trunk (Fig.1D, right one in level 1) enlarged from E (arrow), showing leaf bases.

Scale bars = 1 cm (C), 2 cm (coin diameter, B), 5 cm (D), 27 cm (hammer length).


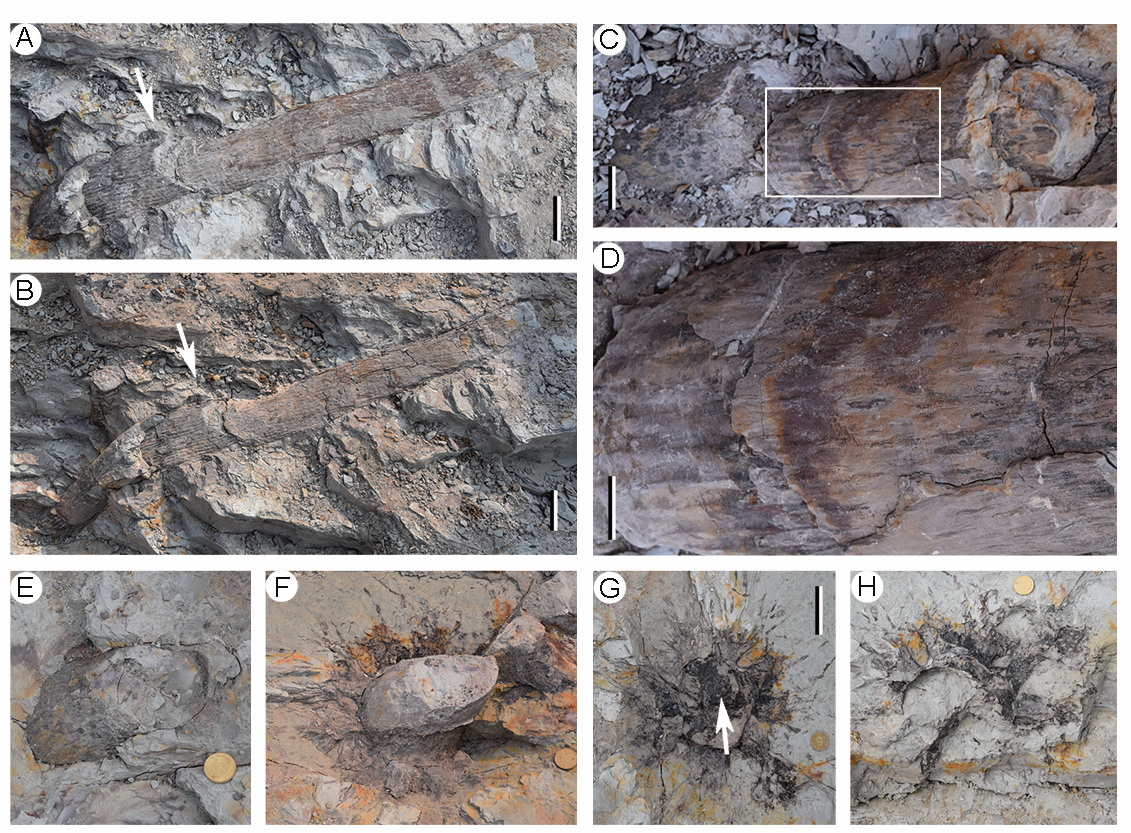


**Fig. S10.** The trunk (arrows in A, B) connected with rooting system (same plant as in Fig. 2A) from level 2 of location 1.

**A**-**H.** Serial excavation of the stem (A-E) and the rooting system (F-H). D showing enlargement of part in C (rectangle). Arrow in g indicating root scars. C, D, PKUB17405.

Scale bars = 1 cm (D), 2 cm (coin diameter, C), 5 cm (A, B, G).

**
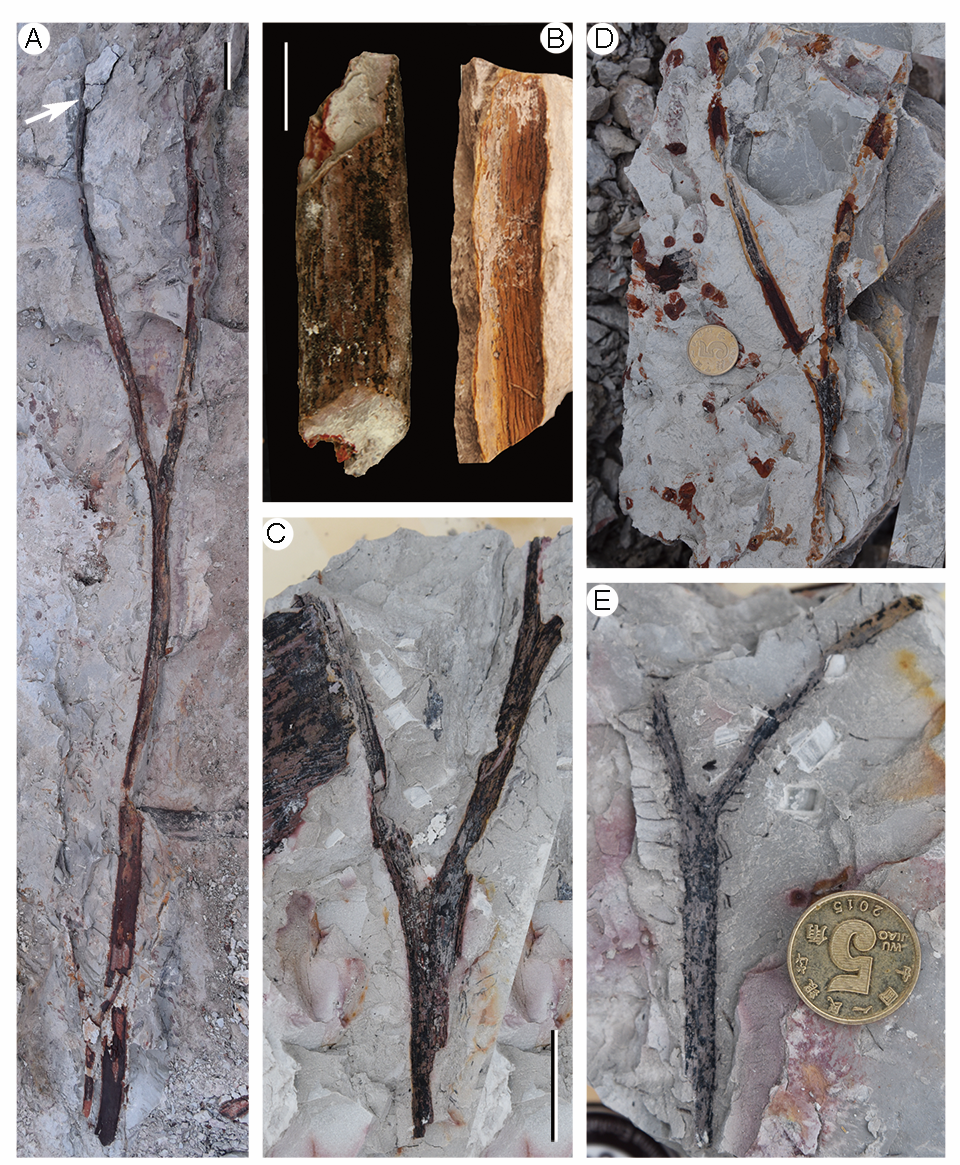
**

**Fig. S11.** Dichotomous branches from location 1.

**A.** A trunk with dichotomy, same as in Fig. 2I, SI-1 Fig. S8A (black arrow).

**B.** Enlargement of removed part in A (arrow), with two sides of an axis showing leaf bases, and right part enlarged in SI-1 Fig. S12J.

**C**-**E.** Dichotomous branches sometimes preserved with leaves. C, PKUB17406.

Scale bars = 1 cm (B), 2 cm (coin diameter, C), 10 cm (A).

**
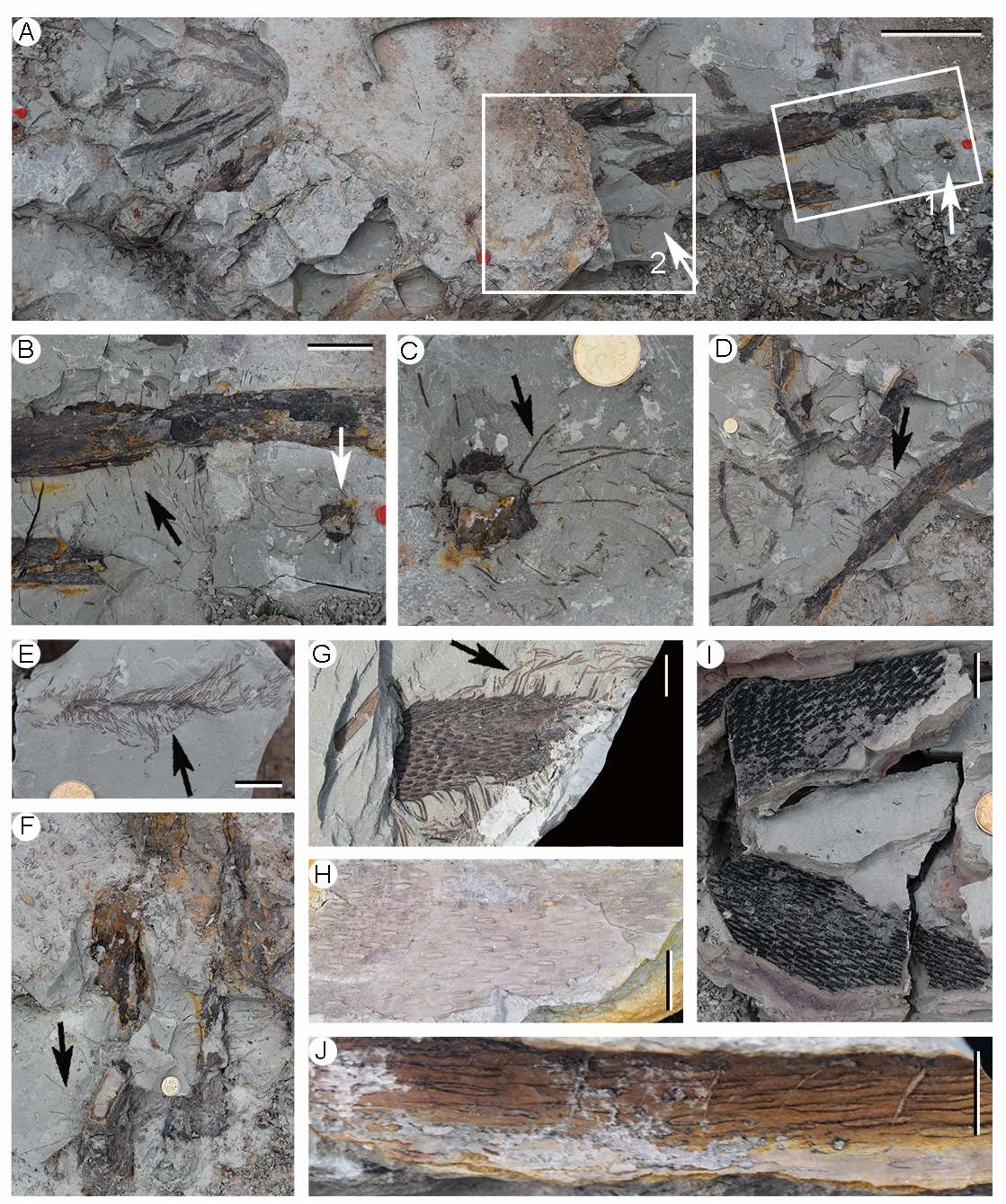
**

**Fig. S12.** The trunks with leaves and /or leaf bases/cushions from location 1.

**A.** Plants from level 2 (excavated in October 2017).

**B.** Enlargement of right rectangle in A, showing trunks with leaves; white arrow indicating the same plant in A (arrow 1), black arrow indicating a leaf.

**C.** Enlargement of part in A (arrow 1) and in B (white arrow), showing a trunk across the bedding plane and with leaves (black arrow). PKUB17407.

**D.** Enlargement of left rectangle in A after excavation, showing stems with leaves (arrow).

**E.** Enlargement of part in A (arrow 2), showing a slender stem with leaves (arrow) parallel to bedding plane. PKUB17408.

**F.** Two trunks with rooting system and leaves from level 2, arrow indicating leaves.

**G**-**J.** Stems with leaf bases. Arrow in G indicating leaves; J showing enlargement of right part in SI-1 Figs. S11B; G, PKUB17409; J, PKUB17410.

Scale bars = 5 mm (J), 1 cm (G, H), 2 cm (coin diameter, E, I), 5 cm (B), 20 cm (A).


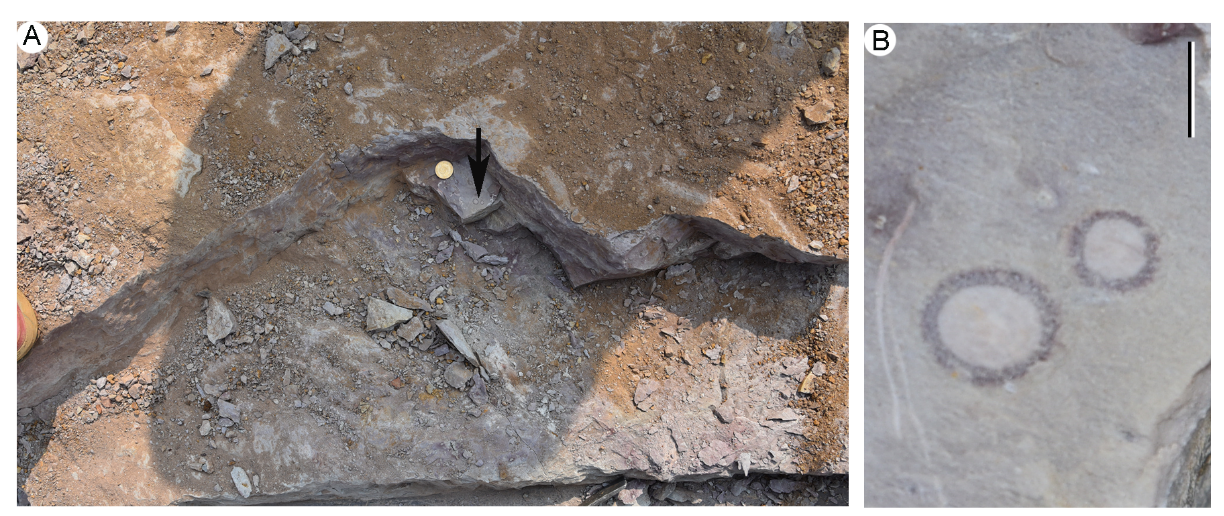


**Fig. S13.** Possible Crinoidea fossils from location 1

**A.** Possible Crinoidea fossils from level 1. Black arrow enlarged in B.

**B.** Enlargement of A (arrow). PKUB17414.

Scale bars = 5 mm (B), 2 cm (coin diameter).

**
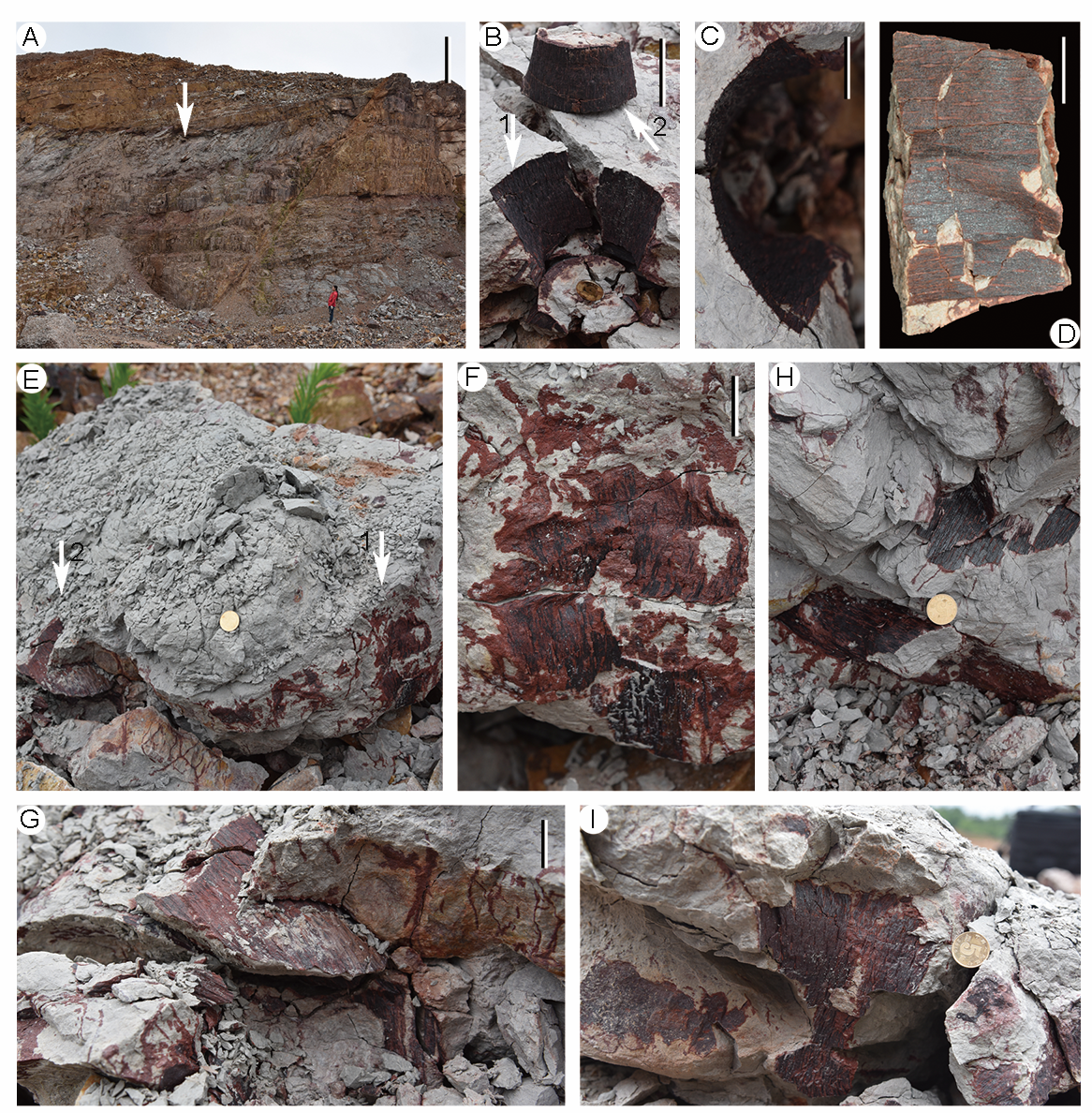
**

**Fig. S14.** Outcrop and *in situ* trunks in location 2.

**A.** Highwall, from which the blocks containing fossil lycopsids fell down (arrow).

**B.** A trunk in fallen block, arrows 1, 2 indicating parts enlarged in C, D, respectively. The picture same as in Fig. 5B.

**C.** Enlargement of part in B (arrow 1), top view of the mould.

**D.** Enlargement of part in B (arrow 2), showing the cast of the trunk with leaf cushions.

**E.** Arrows 1, 2 indicating two trunks in a fallen block, which are enlarged in F, G, respectively. The picture same as in Fig. 5A.

**F, G.** Enlargement of two trunks in E (arrows 1, 2, respectively), which are across the bedding plane.

**H, I.** Two trunks in fallen stone.

Scale bars = 2 cm (coin diameter, C, D, F, G), 5 cm (B), 27 cm (hammer length), 2 m (A).

**
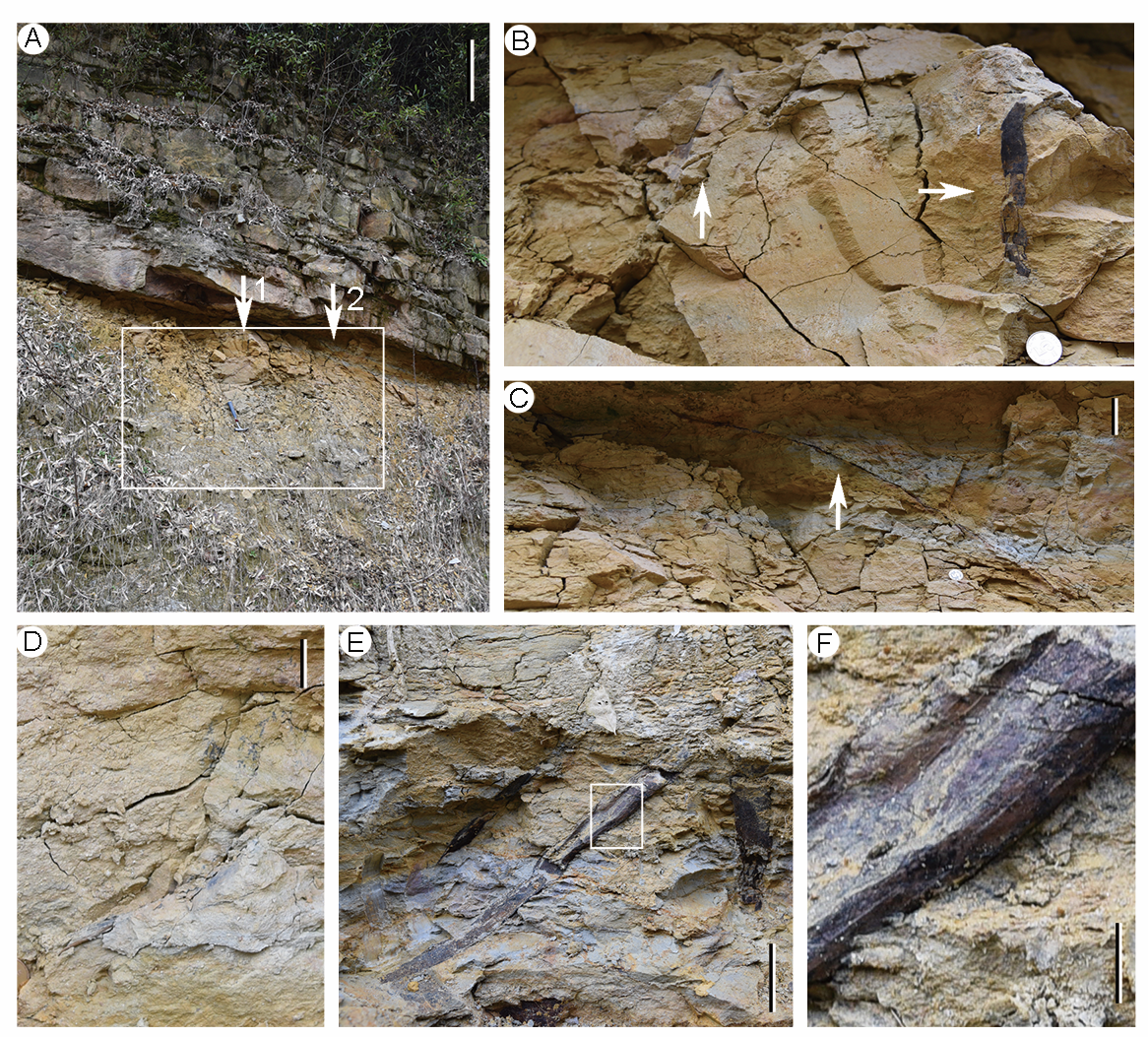
**

**Fig. S15.** Outcrop and *in situ* trunks in location 3.

**A.** Highwall, where the rectangle indicating trunks in B-F before excavation. Arrows 1, 2 indicating areas enlarged in B and C, respectively.

**B.** Two trunks (arrows) in A (arrow 1).

**C.** A trunk (arrow) in A (arrow 2).

**D.** A trunk exposed after excavation of the rectangle area in A.

**E.** Three trunks exposed after excavation of the rectangle area in A. The picture same as in Fig. 5C.

**F.** A trunk with leaf cushions, which is enlarged from the rectangle area in E.

Scale bars = 1 cm (F), 2 cm (coin diameter, D), 5 cm (E, E), 50 cm (A).

**
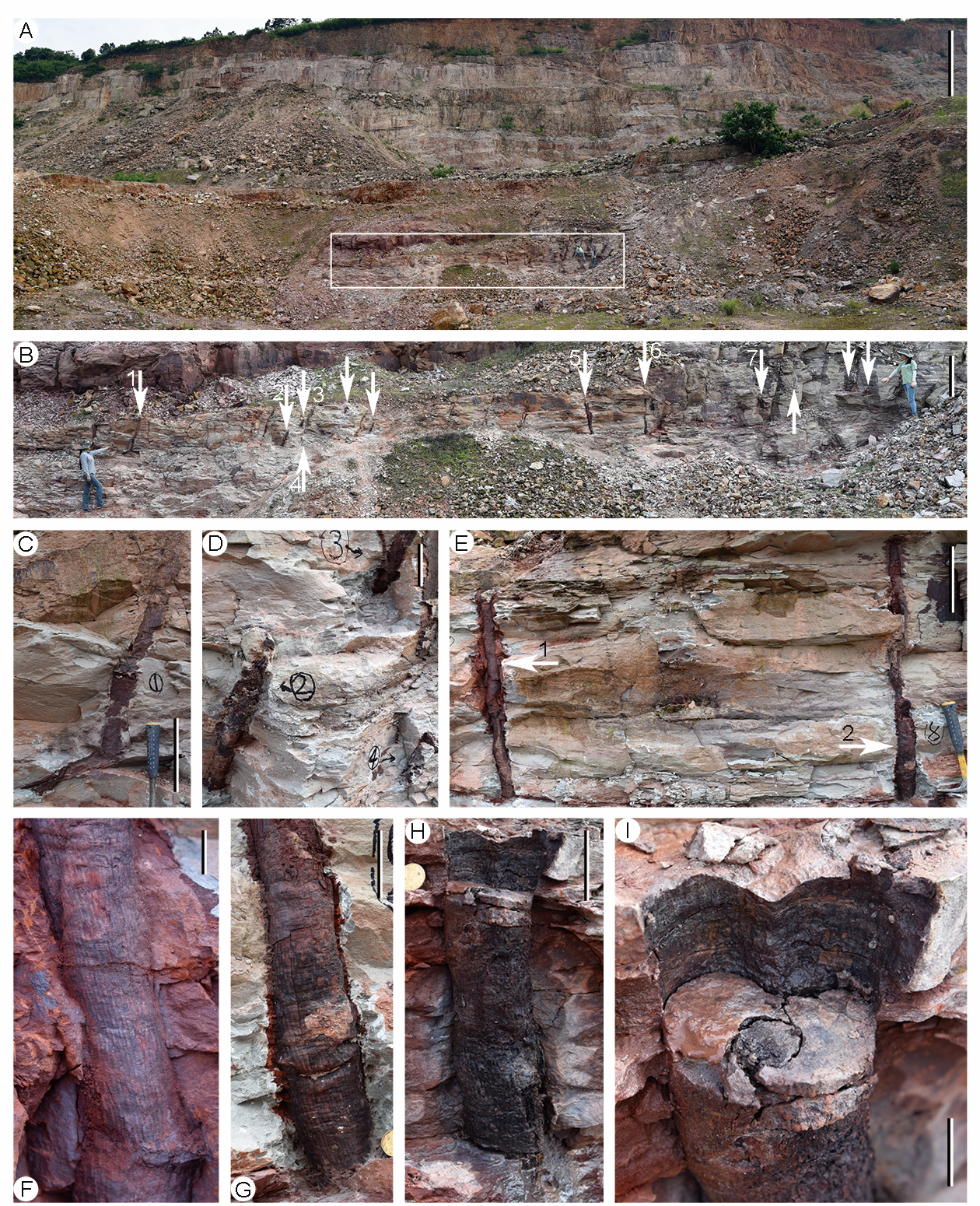
**

**Fig. S16.** Outcrop and *in situ* trunks in location 4.

**A.** Highwall, with the rectangle area being enlarged in B.

**B.** Enlargement of rectangle area in A, showing twelve trunks (arrows), same as in Fig. 5D.

**C.** Enlargement of excavated trunk in B (arrow 1).

**D.** Enlargement of three excavated trunks in B (arrows 2-4).

**E.** Enlargement of two excavated trunks in B (arrows 5, 6).

**F, G.** Stems with leaf cushions, which are enlarged from E (arrows 1, 2, respectively).

**H.** Enlargement of excavated trunk in B (arrow 7).

**I.** Oblique top view of the trunk in H.

Scale bars = 2 cm (F, I), 5 cm (G, H), 10 cm, (D), 20 cm (C, E), 1 m (B), 5 m (A).

**SI-2**


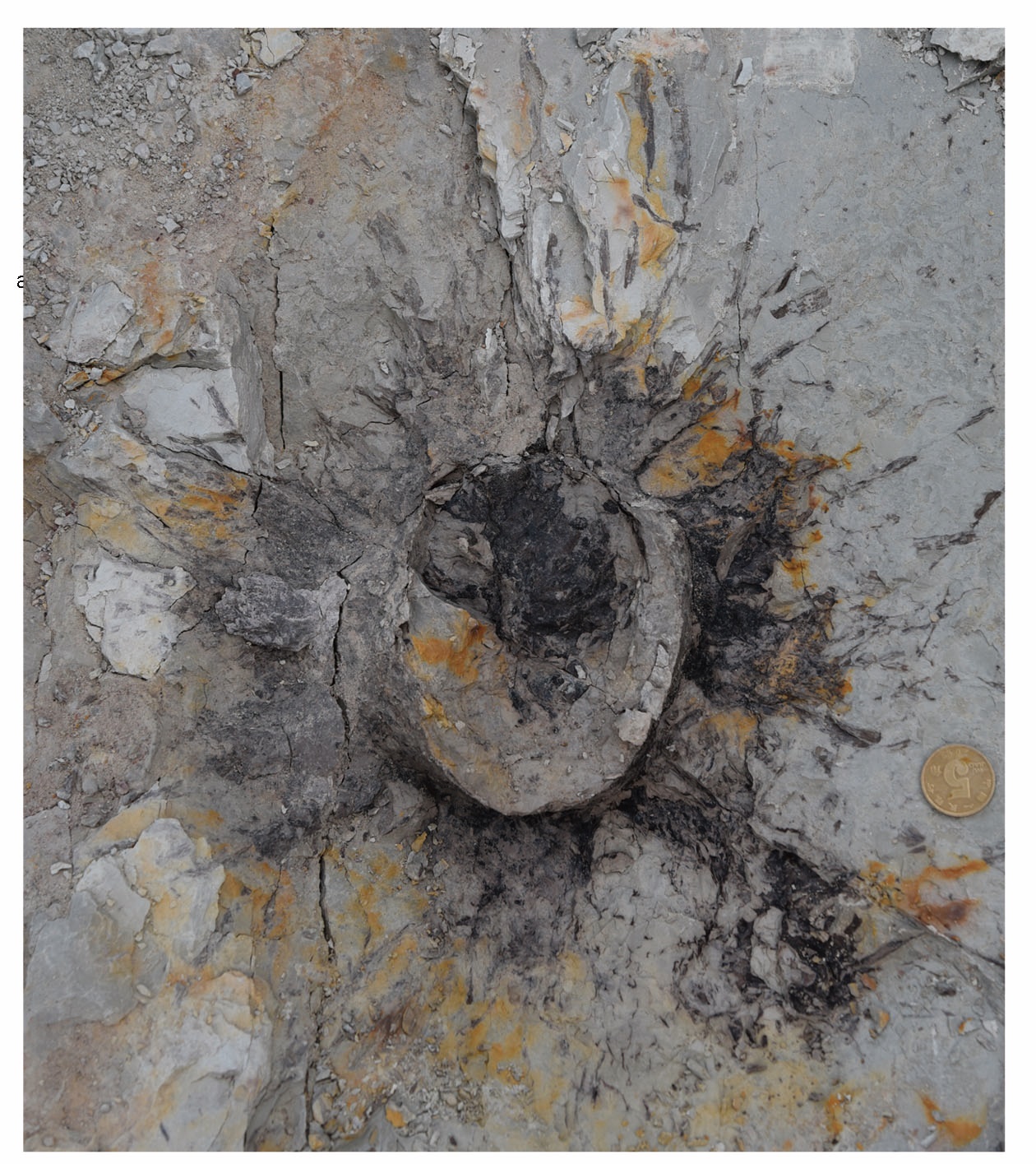


**Fig. S1**. Top view of a rooting system with short rhizomorph lobes, same as Fig. 2A.

Scale bar = 2 cm (coin).


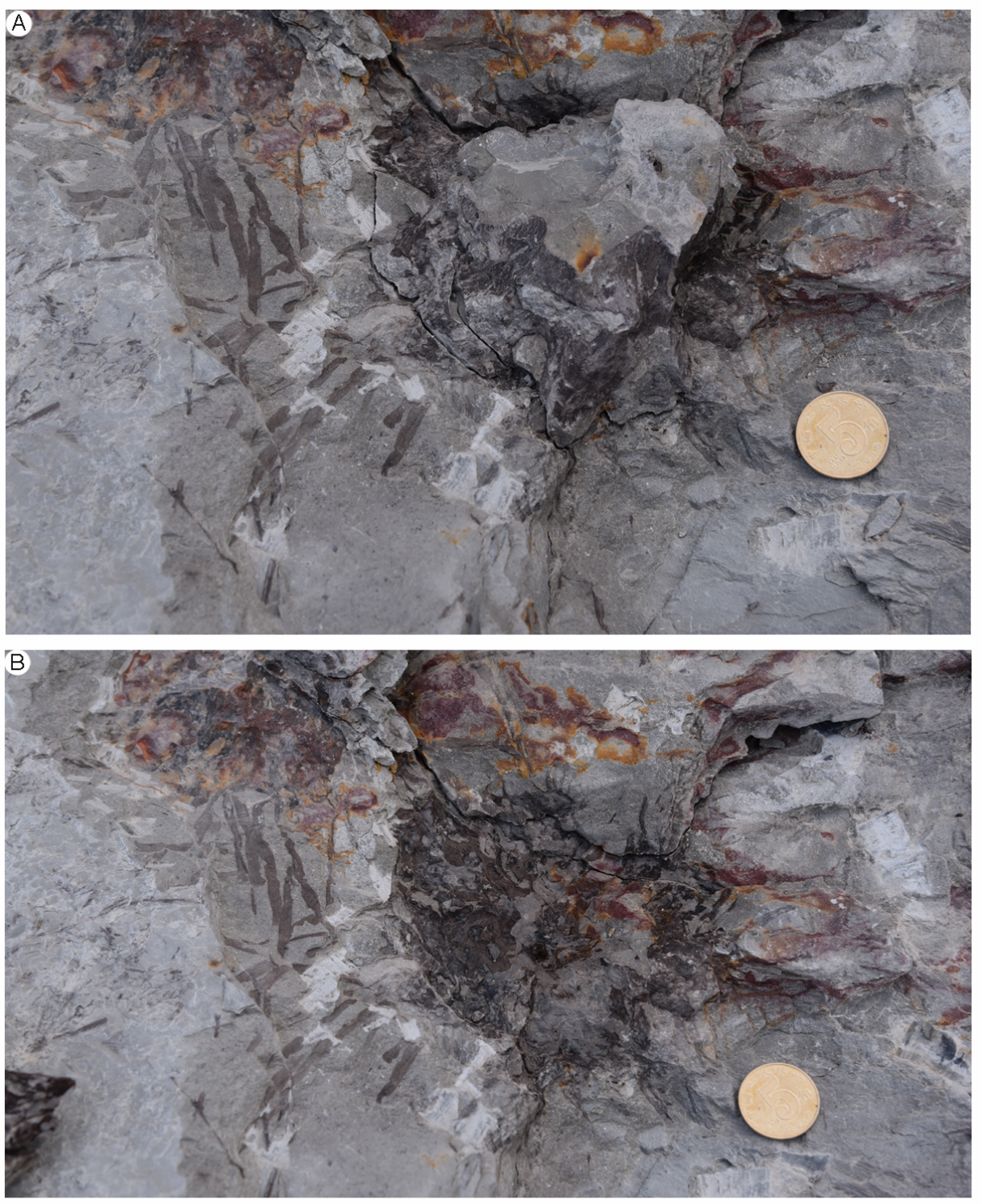


**Fig. S2.** **A**. A trunk with rooting system from level 2, same as Fig. 6C.

**B**. Excavation of the rooting system in A, same as Fig. 6D.

Scale bar = 2 cm (coin).


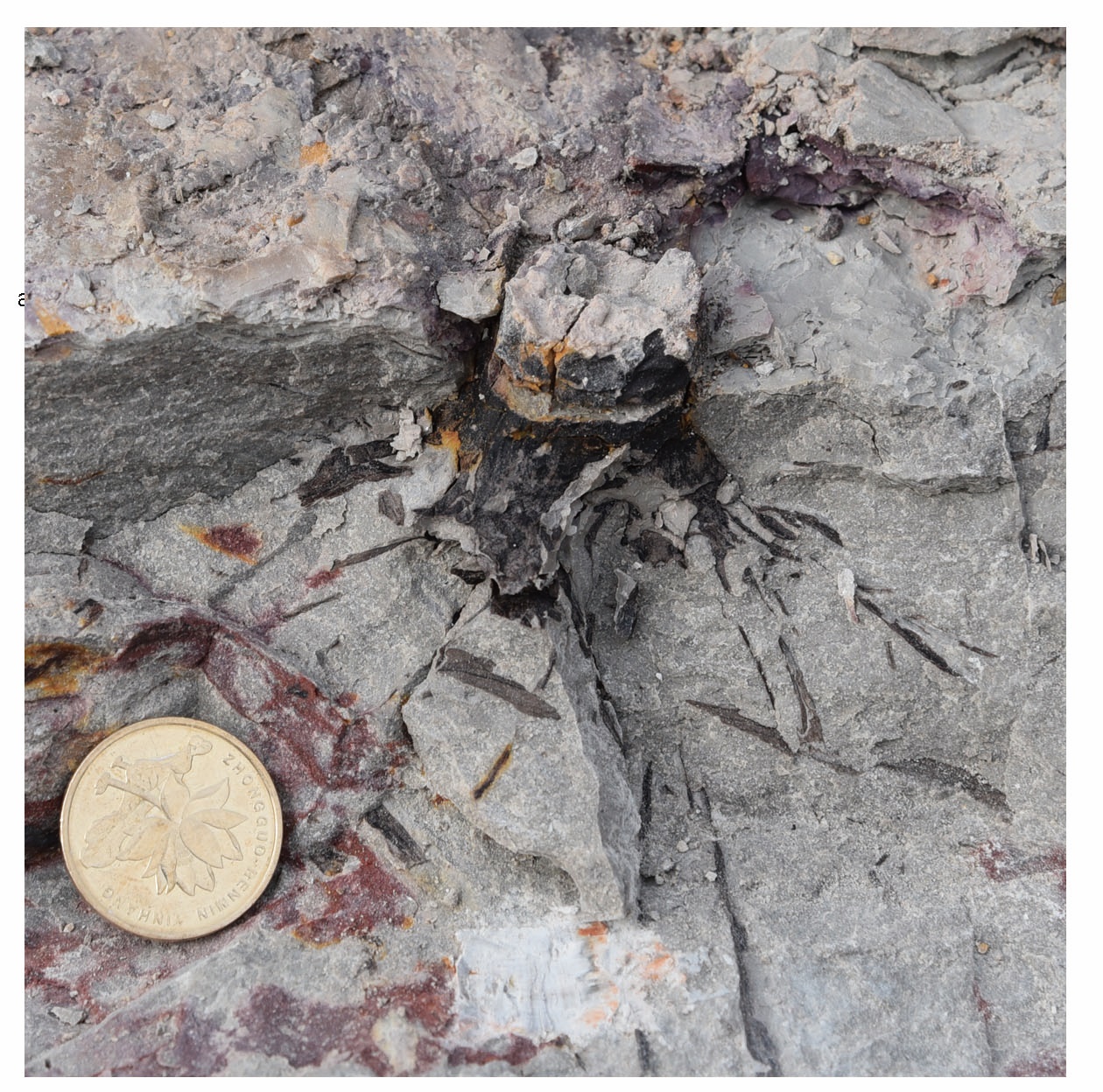


**Fig. S3** Rooting system with short rhizomorh lobes bearing roots, same as SI-1 Fig. S6M.

Scale bar = 2 cm (coin).


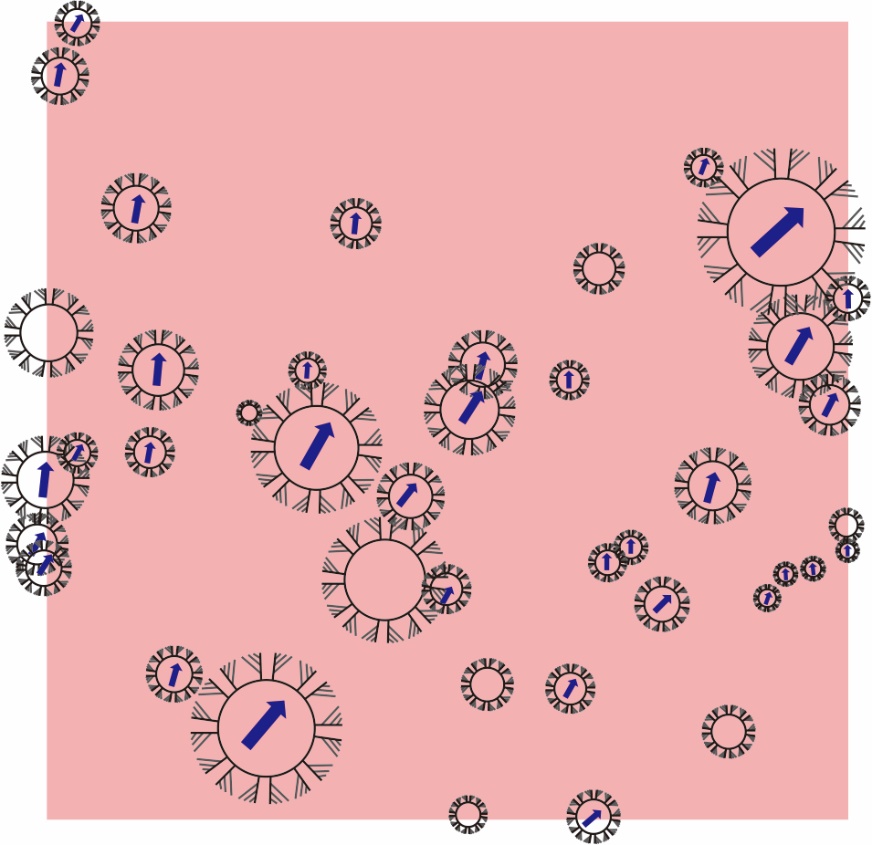


**Fig. S4** Enlargement of pink rectangle (1 m*1 m) in Fig. 1C.
